# Childhood household dysfunction and psychiatric, criminal, and social outcomes in emerging adulthood. A cousin comparison study

**DOI:** 10.1093/ije/dyaf074

**Published:** 2025-05-29

**Authors:** Joonas Pitkänen, Amir Sariaslan, Lauren Bishop, Pekka Martikainen

**Affiliations:** Faculty of Social Sciences, Helsinki Institute for Demography and Population Health, University of Helsinki, Helsinki, Finland; Max Planck—University of Helsinki Research Center for Social Inequalities in Population Health, Helsinki, Finland; Department of Psychiatry, University of Oxford, Oxford, United Kingdom; Faculty of Social Sciences, Helsinki Institute for Demography and Population Health, University of Helsinki, Helsinki, Finland; Max Planck—University of Helsinki Research Center for Social Inequalities in Population Health, Helsinki, Finland; Faculty of Social Sciences, Helsinki Institute for Demography and Population Health, University of Helsinki, Helsinki, Finland; Max Planck—University of Helsinki Research Center for Social Inequalities in Population Health, Helsinki, Finland; Max Planck Institute for Demographic Research, Rostock, Germany

**Keywords:** childhood household dysfunction, psychiatric disorders, substance use, violent crime, property crime, NEET, cousin comparison

## Abstract

**Background:**

Childhood household dysfunction is a well-known risk factor for adverse medical and social outcomes. However, less is known about the extent to which such associations are affected by unmeasured familial confounding.

**Methods:**

This cohort study is based on Finnish register data on birth cohorts 1987–2000 (*n* = 835 987). We considered parental hospital-presenting substance use and psychiatric disorders, prison sentences, death, means-tested social assistance, and union dissolution at ages 0–14 as indicators of childhood household dysfunction. The study participants were followed from age 15 until the end of 2020 for hospital-presenting psychiatric disorders and substance use, psychotropic medication purchases, violent and property crime arrests, and not being in education, employment, or training. The associations were estimated using Cox regression, and cousin comparisons were used to account for unmeasured confounders shared within extended families (*n* = 87 500).

**Results:**

All the exposures were associated with the outcomes in the population-level models, with hazard ratios ranging from 1.3 (95% confidence interval 1.3–1.4) to 2.5 (2.4–2.6). The associations attenuated in the cousin comparisons, on average 12% but with a wide range from −2% to 39% [hazard ratios ranging from 1.2 (1.1–1.4) to 1.9 (1.6–2.3)]. A dose–response relationship between the exposures and the outcomes was observed in the population-level models and the cousin comparisons, with attenuated associations in the latter.

**Conclusion:**

Our findings show systematic associations between childhood household dysfunction and subsequent outcomes. Unobserved confounding likely creates upward bias in these associations, but the extent of this confounding depends on the specific exposure-outcome pairs.

Key Messages

- Previous research has consistently shown associations between childhood household dysfunction and subsequent adverse psychiatric and behavioural outcomes, but the role of shared familial confounders for these associations has been less examined.
- In this cohort study of over 830 000 individuals, after first replicating the well-established associations between household dysfunction and multiple different outcomes at the population level, we found that the vast majority of these associations attenuate to a varying degree (on average 12%) when controlling for shared genetic and environmental factors between full cousins.
- Our findings indicate that part of the associations observed between childhood household dysfunction and subsequent adverse outcomes is explained by unobserved confounding stemming from shared familial factors, but the extent to which depends on specific exposure-outcome pairs.

## Introduction

A large body of literature has documented associations between childhood household dysfunction and health [1–6], behavioural [5, 7, 8], and socioeconomic [9, 10] outcomes. Household dysfunction includes a diverse range of events occurring within the family, such as parental substance use, mental health problems, union dissolution, and incarceration [11]. These experiences commonly co-occur, and a dose–response relationship robust to covariate adjustment between the total number of the indicators and the studied outcomes is typically found [1–3,11].

A central methodological limitation in previous research is that most studies do not assess unobserved confounding in these associations. Such confounding might arise from genetic factors. Studies often include similar indicators measured from both parents and their children, many of which have a genetically heritable component, including psychiatric disorders, substance use disorders, and criminal behaviour [12–15]. Observed parental and offspring indicators may also be expressions of underlying heritable factors, such as personality traits or externalizing disorders [12, 13, 15], and many of the indicators of childhood household dysfunction also have shared genetic determinants [16, 17]. Confounding may also arise from socioeconomic factors that have an intergenerational component [18, 19] and are associated with indicators of household dysfunction [20]. Finally, children may impact parental outcomes [21–23]. For instance, children’s psychiatric diagnoses may be associated with subsequent parental diagnoses, partly due to parental distress [24].

Previous studies on child maltreatment have used, e.g. twin designs [25] and Mendelian randomization [26] to adjust for unobserved familial confounding, whereas studies on household dysfunction indicators, such as parental divorce or mental health problems, have addressed this issue using, e.g. children-of-twins and adoption designs [27–30], or molecular genetics designs, such as polygenic risk scores together with single nucleotide polymorphism based heritability [22, 30]. The results from these studies indicate that these experiences have a direct association with offspring mental health outcomes, net of genetic confounding [30]. However, these associations depend on specific exposure-outcome pairs, e.g. the association between parental mental health problems and offspring mental health problems is less confounded than the associations between parental criminality and offspring mental health [22], and parental divorce is an environmental risk factor for externalizing but not internalizing disorders [27].

A common approach of adjusting for unobserved confounding is also a sibling fixed effects design, which accounts for an average of 50% of co-segregating genes and the environment shared by the siblings [31]. Several studies have used this approach to study childhood household dysfunction and showed that the associations between the exposures and outcomes largely attenuate in sibling comparisons [32–34]. Similar findings have been reported in studies using sibling comparisons to study child maltreatment [25] and adverse childhood experiences, a combination of maltreatment and household dysfunction [35–37].

However, a key caveat of the sibling fixed effects design is that the estimates are based only on information from siblings who are differentially exposed to the indicator of interest [31], but many of the childhood household dysfunction indicators are typically shared between siblings, especially if they are measured as ever experienced during childhood. A workaround for this problem is to compare outcomes between discordantly exposed cousins. This design accounts for 12.5% of the co-segregating genes of the cousins and, in addition, for those environmental factors that are shared by the cousins [38, 39]. Cousin comparisons have not been widely used to study the outcomes of multiple household dysfunction indicators, except for three Swedish studies, which showed that the population-level estimates of the associations between household dysfunction and neurodevelopmental disorders [32], substance use [34], and drug use [40] attenuate between 20% and 30% [32, 34] or more [40] in cousin comparisons.

In this study, we use cousin comparisons to assess unobserved confounding in the associations between childhood household dysfunction and multiple outcomes in emerging adulthood. Based on previous research on childhood adversities [1–3, 11] and data availability, we included parental psychiatric and substance-attributable hospitalizations, death, prison sentence, union dissolution, and social assistance as household dysfunction indicators. We assess six outcomes in emerging adulthood, which we expect to be associated with household dysfunction based on previous research [1, 8, 10]: hospital-presenting psychiatric disorders and substance use, psychotropic medication, not being in education, employment or training (NEET), and violent and property crime arrests. We assess the associations between single household dysfunction indicators and the outcomes since the amount of unobserved confounding may differ by specific exposure-outcome constellations [22]. We expect there to be unobserved familial confounding in all the associations, but more in the associations between similar parental and offspring variables. We also study the well-known dose–response relationship between the parental indicators and the outcomes. Given the accumulation of multiple exposures with intergenerational associations, we expect to find more unobserved confounding in the associations between multiple indicators of household dysfunction and the outcomes than between single exposures and the outcomes.

## Data

The study is based on register data on all individuals born in Finland in 1987–2000 and residing in Finland at age 14 (*n* = 854 082). We excluded those without full observations between ages 0–14 (*n* = 9049) and those without information on biological parents at birth (*n *= 9046). The final analytical sample size was 835 987. To identify cousins, we included the first-born children of each mother in the data, and defined cousins as sharing the same maternal grandparents but having a different mother. In total, we identified 87 500 first-born full cousins, nested within 40 898 maternal grandparents.

For all the parents and their children, annual information on sociodemographic variables, mortality, police-reported crime, and criminal convictions was obtained from Statistics Finland. These data were linked with inpatient hospital episodes and specialized outpatient visits from the Finnish Institute for Health and Welfare and psychotropic medication purchases from the Social Insurance Institution of Finland.

## Methods

### Household dysfunction

We included parental substance-attributable and psychiatric inpatient episodes, death of either parent, unconditional prison sentences, union dissolutions, and social assistance (a means-tested last-resort financial assistance) as indicators of household dysfunction. All indicators were measured as ever experienced between ages 0–14, using data from both biological mothers and fathers. Detailed descriptions of the data sources and definitions are available in Supplementary Table S1.

### Outcomes

Hospital-presenting substance use and psychiatric disorders, violent and property crime arrests, psychotropic medication purchases, and being NEET for at least 2 consecutive years were considered as outcomes in emerging adulthood (Supplementary Table S2). We followed the study participants for these outcomes from 1st January of the year they turned 15 years old until the date of the outcome, death, or emigration or the end of 2020 (2019 for medication purchases), when the participants were aged 20 to 33. For NEET, exact months and days of the event were unavailable; therefore, we used the last day of the first NEET year of the 2-year NEET period as the event date. We had labour market data until 2019, but since we defined NEET as consisting of at least 2 consecutive NEET years, we ended the follow-up for this outcome in 2018 as no one could become NEET in 2019 with this definition.

### Observed confounders

Statistics Finland’s data on parental education, region of residence, birth order, maternal age at birth, sex, and an indicator for two-parent family were included as observed confounders (Supplementary Table S3). These variables were measured at age 0.

### Modelling approach

#### Single indicators of household dysfunction

We used Cox regression to examine the associations between each indicator of childhood household dysfunction and each outcome. For every association, we fitted a crude model, and a model adjusted for observed confounders using the population-level data. Cluster-robust standard errors were used to account for correlation between full siblings.

We then used the data on the 87 500 first-born full cousins and conducted cousin comparisons with stratified Cox models. The cousins within each grandparent dyad shared the same baseline hazard and were compared to each other only. The estimated associations were mostly derived from discordantly exposed cousins and thus the number of informative strata differs by exposure. The cousin comparisons were adjusted for all observed confounders except birth order, which did not vary between the cousins.

#### Accumulation of household dysfunction

We assessed the accumulation of the household dysfunction indicators by summing up all the binary indicators, with a cut-off at four. The sum score was used as a categorical covariate in the confounder-adjusted models, both in total population and in the cousin comparison framework. All the analyses were conducted with Stata 17.

#### Additional analyses

To assess whether our results are driven by prevalent outcomes, we repeated our main analyses by excluding individuals whose first outcome occurred before the start of follow-up (age 15). We also examined the co-occurrence of the household dysfunction indicators by calculating a tetrachoric correlation matrix of the variables, and then by conducting an exploratory factor analysis (EFA) on this matrix.

## Results

### Descriptive characteristics

Parental union dissolution and social assistance were the most common indicators of household dysfunction (Table 1). Of the outcomes, psychotropic medication purchases were the most common (26%), followed by 2-year NEET (23%) and hospital-presenting psychiatric disorders (17%) (Supplementary Table S4). All outcomes were more common among those exposed to household dysfunction (Table 1, Supplementary Table S4). Outcome rates and proportions by confounders are presented in Supplementary Tables S5 and S6. The descriptive characteristics were similar in the cousin population (Supplementary Tables S7–S10), except that the mothers were younger due to the inclusion of only first-borns.

**Table 1.** Distribution of the household dysfunction indicators and rates of outcomes (per 1000 person-years).

		Offspring outcome, rate/1000 person-years (95% CI)					
Household dysfunction indicators (measured from parents at child’s ages 0–14 years)	Total distribution, n (%)	Hospital-presenting psychiatric disorder	Hospital-presenting substance use	Psychotropic medication purchases	2-year NEET	Violent crime arrest	Property crime arrest
Parental psychiatric hospitalization							
No	784 405 (94)	13.9 (13.8–14.0)	3.9 (3.9–4.0)	24.7 (24.6–24.8)	23.8 (23.7–24.0)	5.0 (5.0–5.1)	8.6 (8.5–8.6)
Yes	51 582 (6)	28.7 (28.2–29.2)	8.3 (8.1–8.5)	44.1 (43.5–44.8)	37.9 (37.3–38.5)	9.4 (9.1–9.7)	16.1 (15.7–16.5)
Parental substance-attributable hospitalization							
No	793 904 (95)	14.1 (14.0–14.2)	3.9 (3.8–3.9)	24.9 (24.8–25.0)	23.7 (23.6–23.8)	4.9 (4.9–5.0)	8.4 (8.3–8.5)
Yes	42 083 (5)	27.3 (26.7–27.8)	10.5 (10.2–10.8)	43.5 (42.8–44.2)	43.9 (43.2–44.6)	12.9 (12.6–13.3)	21.3 (20.9–21.8)
Parental death							
No	810 842 (97)	14.5 (14.4–14.5)	4.1 (4.0–4.1)	25.5 (25.3–25.6)	24.3 (24.2–24.4)	5.2 (5.1–5.2)	8.8 (8.7–8.9)
Yes	25 145 (3)	23.1 (22.5–23.7)	7.7 (7.4–8.1)	36.2 (35.4–37.0)	36.6 (35.8–37.4)	9.2 (8.9–9.6)	15.5 (15.0–16.0)
Parental prison sentence							
No	821 564 (98)	14.5 (14.4–14.6)	4.0 (4.0–4.1)	25.4 (25.3–25.5)	24.1 (24.0–24.2)	5.0 (5.0–5.1)	8.6 (8.5–8.7)
Yes	14 423 (2)	29.6 (28.7–30.5)	14.8 (14.2–15.5)	50.0 (48.7–51.3)	60.7 (59.2–62.2)	22.8 (21.9–23.6)	37.3 (36.1–38.6)
Parental union dissolution							
No	581 578 (70)	12.2 (12.1–12.3)	3.2 (3.1–3.2)	22.3. (22.2–22.5)	21.2 (21.1–21.4)	4.0 (3.9–4.0)	6.7 (6.7–6.8)
Yes	254 409 (30)	20.9 (20.7–21.1)	6.6 (6.5–6.7)	34.4 (34.1–34.6)	33.1 (32.9–33.4)	8.4 (8.3–8.6)	14.7 (14.6–14.9)
Parental social assistance							
No	549 403 (66)	11.5 (11.4–11.6)	2.8 (2.7–2.8)	21.3 (21.2–21.4)	18.0 (17.8–18.1)	3.2 (3.1–3.2)	5.6 (5.6–5.7)
Yes	286 584 (34)	21.2 (21.0–21.4)	6.9 (6.8–7.0)	34.8 (34.6–35.1)	38.5 (38.3–38.8)	9.5 (9.4–9.6)	15.9 (15.8–16.1)
Number of parental indicators							
0	431 740 (52)	10.4 (10.3–10.5)	2.4 (2.4–2.5)	19.8 (19.7–19.9)	17.1 (17.0–17.3)	2.8 (2.8–2.9)	5.0 (4.9–5.1)
1	214 391 (26)	15.8 (15.6–16.0)	4.4 (4.3–4.5)	27.4 (27.1–27.6)	27.2 (27.0–27.4)	5.6 (5.5–5.7)	9.6 (9.5–9.7)
2	134 481 (16)	22.0 (21.7–22.2)	6.9 (6.8–7.1)	36.0 (35.7–36.4)	38.1 (37.7–38.5)	9.6 (9.4–9.7)	16.5 (16.3–16.8)
3	35 254 (4)	28.4 (27.8–29.0)	9.8 (9.5–10.1)	44.3 (43.6–45.1)	45.0 (44.3–45.8)	12.8 (12.4–13.2)	21.7 (21.2–22.3)
4 or more	20 121 (2)	33.2 (32.3–34.1)	13.2 (12.7–13.7)	51.4 (50.3–52.6)	51.1 (50.0–52.2)	15.9 (15.4–16.5)	26.5 (25.7–27.3)

CI: confidence interval; NEET: not in education, employment, or training.

**Figure 1.**
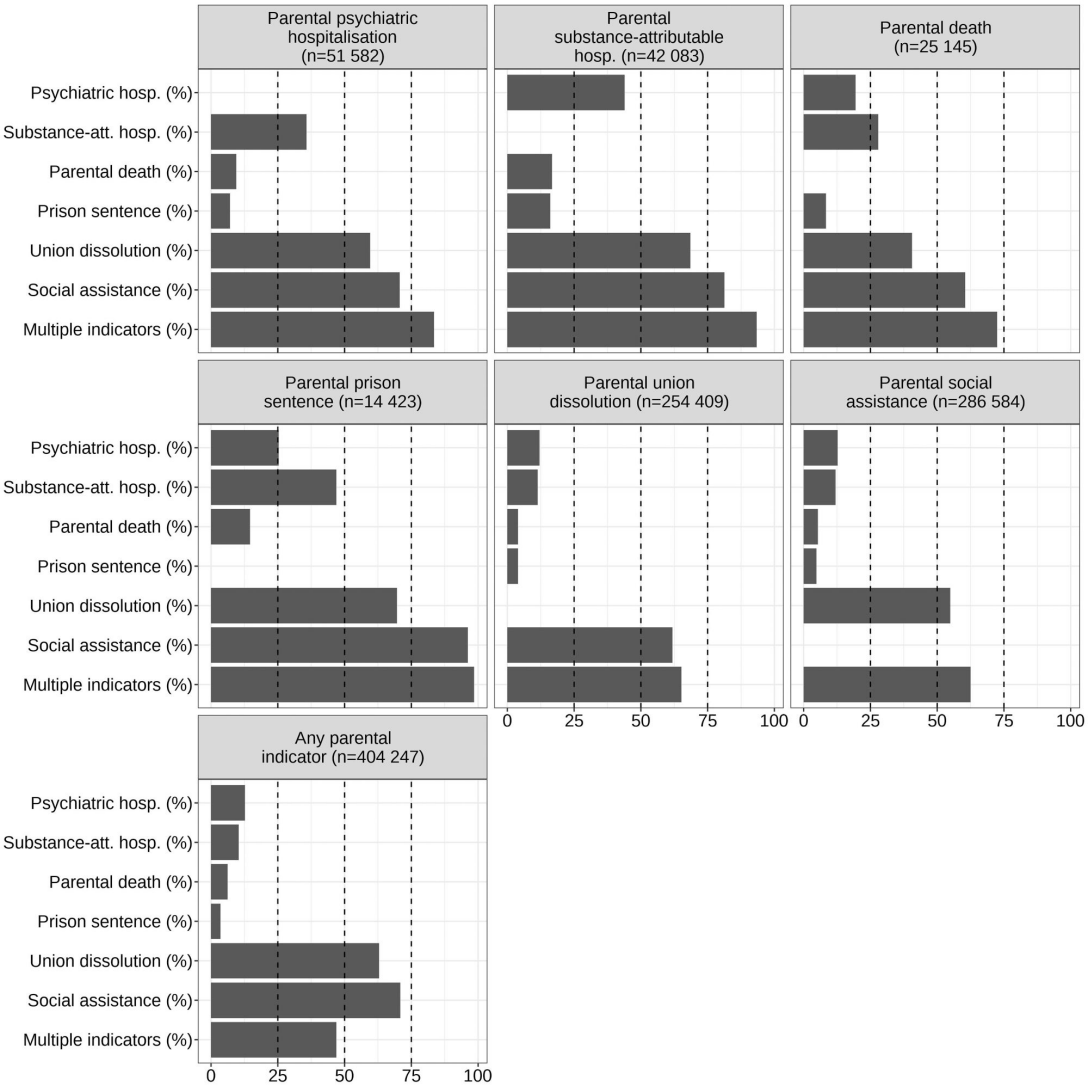
The co-occurrence of parental household dysfunction indicators. The bars show the percentage of individuals with the indicator and the percentage of those with multiple indicators if exposed to the indicator shown in the panel title. The lowest panel shows the prevalence of all the indicators and the prevalence of multiple indicators among those exposed to at least one. Calculated from the total population data (*n* = 835 987).

**Figure 2.**
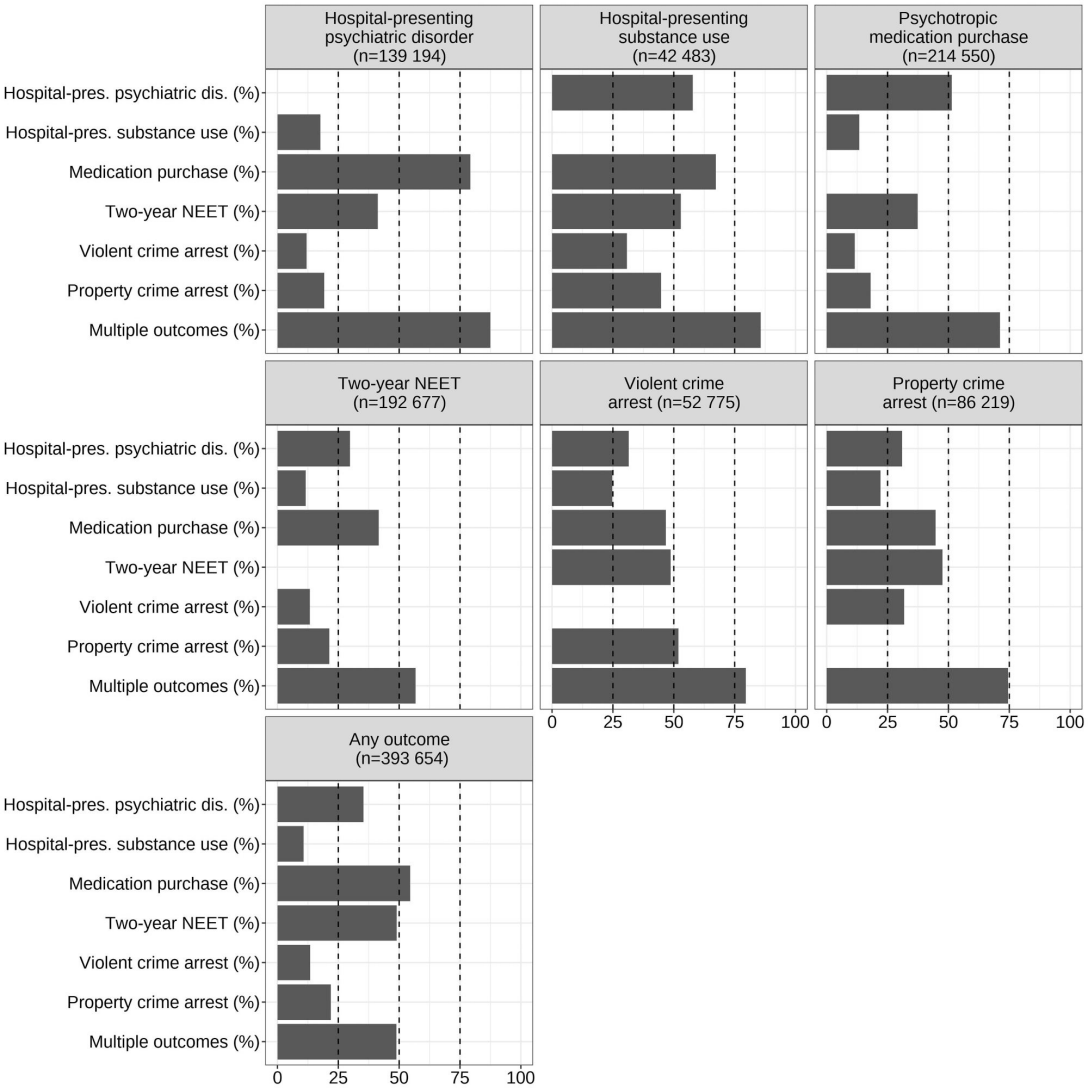
The co-occurrence of offspring outcomes. The bars show the percentage of individuals with the outcome and the percentage of those with multiple outcomes if the individual has the outcome shown in the panel title. The lowest panel shows the prevalence of all the outcomes and the prevalence of multiple indicators among those with at least one. Calculated from the total population data (*n* = 835 987).

Both the exposures and outcomes had overlaps (Figs 1 and 2). Forty-seven percent of individuals experiencing any of the household dysfunction indicators had at least two, and 49% of those with any of the outcomes had at least two. The most common co-occurring household dysfunction indicators were social assistance and union dissolution (Fig. 1), and the most common co-occurring outcomes were psychotropic medication purchases, NEET, and hospital-presenting psychiatric disorders (Fig. 2).

**Figure 3.**
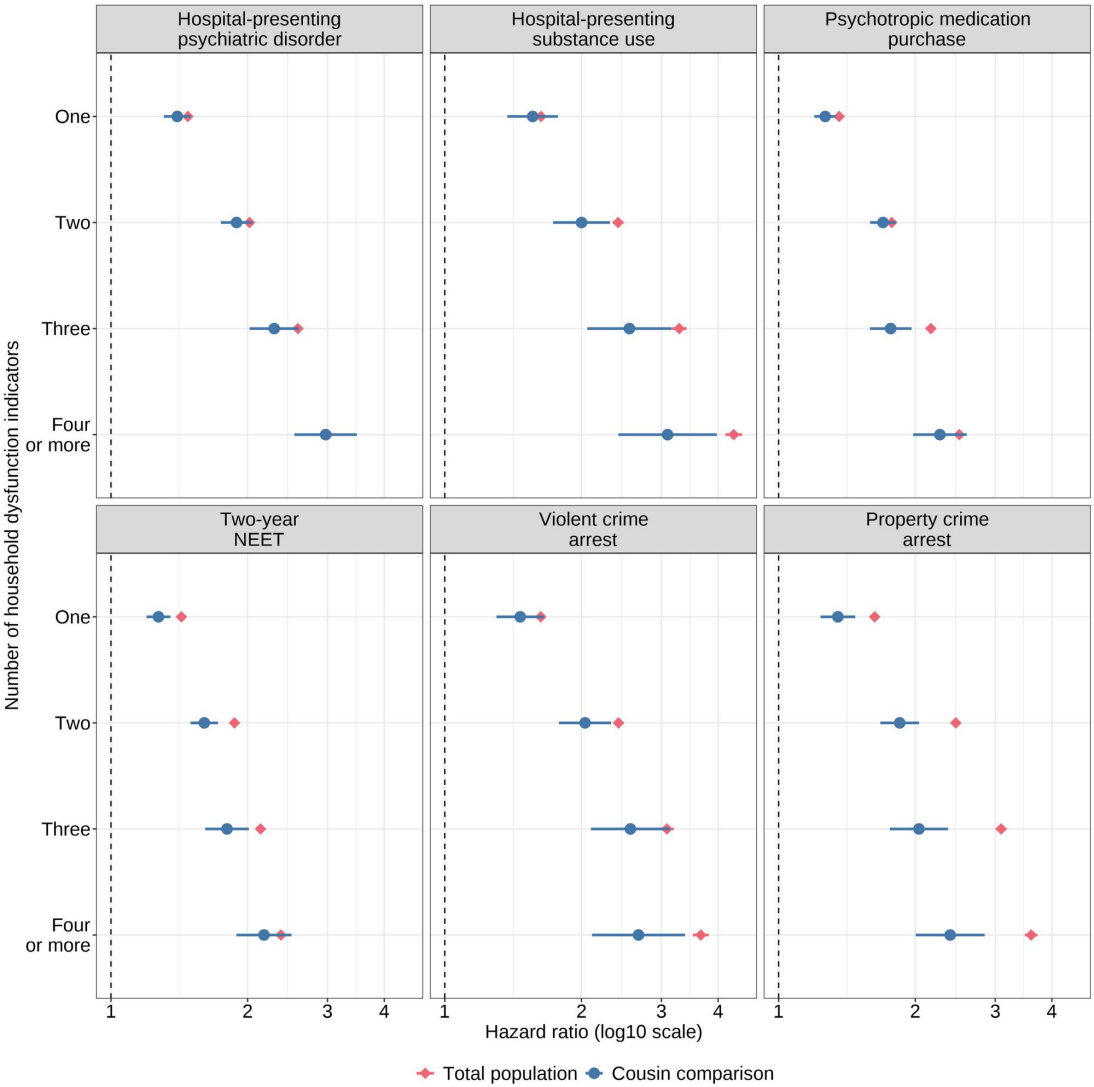
Associations between the sum score of indicators of household dysfunction and psychiatric, criminal, and social outcomes in the total population and in the cousin comparison. Models are adjusted for parental education, region of residence, birth order, maternal age, child’s sex, and an indicator for two-parent family at age 0. Hazard ratios and 95% confidence intervals are plotted on a log scale. Note that in the total population model, the confidence intervals are narrow and therefore not clearly visible. NEET: not in education, employment, or training.

### Single indicators of household dysfunction

#### Parental psychiatric hospitalization

Parental psychiatric hospitalization was associated with increased hazards of all the outcomes in the population-level models, with hazard ratios (HRs) ranging between 1.4 and 1.9 (Table 2, *P*-values in Supplementary Table S11). The covariate-adjusted associations were attenuated by 6%–15% (on average 9%) in the cousin comparison. The largest attenuation was observed for crime-related outcomes and psychotropic medication (Table 2, Supplementary Table S12). The 95% confidence intervals (CIs) from the cousin comparison overlapped with the intervals from population-level covariate-adjusted models for all outcomes, except psychotropic medication purchases.

**Table 2.** Associations between single indicators of household dysfunction and psychiatric, criminal, and social outcomes in emerging adulthood: results from crude and confounder-adjusted models in the total population and confounder-adjusted models in the cousin comparison.

		Offspring outcome, hazard ratio (95% CI)					
Household dysfunction indicators (measured at child’s ages 0–14 years)	Model sample size	Hospital-presenting psychiatric disorder	Hospital-presenting substance use	Psychotropic medication purchases	2-year NEET	Violent crime arrest	Property crime arrest
Parental psychiatric hospitalization							
Crude	835 987	2.05 (2.01–2.09)	2.11 (2.04–2.17)	1.80 (1.77–1.83)	1.61 (1.58–1.64)	1.85 (1.80–1.91)	1.84 (1.80–1.88)
Adjusted^a^	835 987	1.91 (1.88–1.95)	1.81 (1.75–1.87)	1.70 (1.67–1.73)	1.40 (1.37–1.42)	1.51 (1.46–1.55)	1.54 (1.50–1.57)
Cousin comparison^a^	87 500^b^	1.81 (1.64–1.99)	1.70 (1.43–2.03)	1.52 (1.39–1.65)	1.31 (1.20–1.43)	1.28 (1.09–1.51)	1.34 (1.19–1.51)
Parental substance-attributable hospitalization							
Crude	835 987	1.93 (1.89–1.97)	2.73 (2.65–2.81)	1.75 (1.72–1.78)	1.88 (1.85–1.92)	2.63 (2.56–2.71)	2.49 (2.44–2.55)
Adjusted	835 987	1.74 (1.71–1.78)	2.20 (2.14–2.27)	1.62 (1.59–1.65)	1.53 (1.50–1.55)	1.93 (1.87–1.98)	1.89 (1.85–1.93)
Cousin comparison	87 500	1.62 (1.45–1.81)	1.92 (1.61–2.30)	1.49 (1.35–1.64)	1.47 (1.33–1.62)	1.69 (1.43–2.00)	1.49 (1.32–1.69)
Parental death							
Crude	835 987	1.59 (1.55–1.64)	1.90 (1.82–1.99)	1.42 (1.39–1.45)	1.52 (1.48–1.55)	1.80 (1.73–1.87)	1.76 (1.70–1.82)
Adjusted	835 987	1.49 (1.45–1.53)	1.67 (1.60–1.75)	1.35 (1.32–1.38)	1.31 (1.28–1.35)	1.49 (1.43–1.55)	1.49 (1.44–1.54)
Cousin comparison	87 500	1.43 (1.23–1.66)	1.35 (1.06–1.72)	1.33 (1.17–1.52)	1.23 (1.07–1.41)	1.51 (1.18–1.93)	1.47 (1.23–1.76)
Parental prison sentence							
Crude	835 987	2.03 (1.97–2.10)	3.70 (3.55–3.86)	1.98 (1.93–2.03)	2.62 (2.55–2.69)	4.47 (4.31–4.64)	4.07 (3.95–4.20)
Adjusted	835 987	1.59 (1.54–1.64)	2.37 (2.27–2.48)	1.61 (1.57–1.66)	1.79 (1.74–1.84)	2.51 (2.41–2.61)	2.38 (2.30–2.46)
Cousin comparison	87 500	1.57 (1.33–1.86)	1.44 (1.13–1.84)	1.34 (1.16–1.55)	1.61 (1.39–1.86)	1.76 (1.40–2.21)	1.48 (1.25–1.76)
Parental union dissolution							
Crude	835 987	1.70 (1.68–1.72)	2.08 (2.04–2.13)	1.55 (1.53–1.56)	1.58 (1.56–1.60)	2.09 (2.05–2.13)	2.11 (2.08–2.14)
Adjusted	835 987	1.60 (1.58–1.62)	1.85 (1.81–1.89)	1.48 (1.47–1.50)	1.44 (1.43–1.46)	1.78 (1.74–1.81)	1.83 (1.80–1.85)
Cousin comparison	87 500	1.50 (1.42–1.59)	1.45 (1.32–1.61)	1.38 (1.31–1.44)	1.29 (1.23–1.36)	1.54 (1.40–1.69)	1.44 (1.34–1.55)
Parental social assistance							
Crude	835 987	1.84 (1.82–1.86)	2.50 (2.45–2.55)	1.64 (1.62–1.65)	2.17 (2.15–2.19)	3.01 (2.95–3.06)	2.79 (2.75–2.83)
Adjusted	835 987	1.77 (1.75–1.79)	2.08 (2.04–2.13)	1.59 (1.57–1.60)	1.77 (1.75–1.79)	2.11 (2.07–2.16)	2.09 (2.06–2.12)
Cousin comparison	87 500	1.61 (1.51–1.72)	1.77 (1.57–1.99)	1.45 (1.38–1.53)	1.56 (1.47–1.65)	1.81 (1.62–2.02)	1.63 (1.50–1.77)

a All confounder-adjusted models and cousin comparisons were adjusted for parental education, region of residence, birth order, maternal age, child’s sex, and an indicator for two-parent family. These covariates were measured at age 0.

b Number of individuals who share maternal grandparents with at least one other individual in the data. Only first-born children of each mother are included. Number of informative strata differs across exposures and outcomes.

CI: confidence interval; NEET: not in education, employment, or training.

#### Parental substance-attributable hospitalization

The HRs varied between 1.5 and 2.2 in the population-level covariate-adjusted models (Table 1). The estimates were attenuated by 4%–21% (on average 11%) in the cousin comparison, the most for offspring property crime arrest (Table 2, Supplementary Table S12). This association was the only one where the CIs of the HRs from the cousin comparisons did not overlap with the population level CIs.

#### Parental death

The HRs ranged between 1.3 and 1.7 in the covariate-adjusted population models (Table 2). In the cousin comparison, the effect sizes were of similar magnitude for most of the outcomes (Table 2). The largest attenuation (19%) was observed for hospital-presenting substance use (Supplementary Table S12). The CIs between the cousin comparison and the covariate-adjusted population model overlapped in all the associations.

#### Parental prison sentence

In the population-level adjusted model, the HRs ranged between 1.6 and 2.5 (Table 2). In cousin comparisons, the hazards were attenuated by 1%–39%, on average 22%. The largest attenuations were observed for offspring substance use and crime-related outcomes, followed by psychotropic medication purchases. In these associations, there was no overlap in CIs between the population-level model and the cousin comparison. In general, both the effect sizes and attenuation after adjustment for unobserved confounding were the largest for this exposure.

#### Parental union dissolution

This exposure was associated with 40%–85% increased risk of the outcomes in the adjusted population-level model. These estimates were attenuated by 6%–21% (on average 13%) in the cousin comparisons, most for hospital-presenting substance use and property crime. The 95% CIs from population-level model and cousin comparisons only overlapped in the association between the exposure and offspring hospital-presenting psychiatric disorders.

#### Parental social assistance

The HRs for social assistance ranged between 1.6 and 2.1 in the covariate-adjusted models. These estimates were attenuated by 9%–22% (on average 14%) in the cousin comparison, the most for offspring property crime. The 95% CIs from population-level model and cousin comparisons only overlapped in the association between social assistance and offspring hospital-presenting psychiatric disorders.

### Accumulation of household dysfunction

A gradient between the number of household dysfunction indicators and the hazards for all outcomes was observed at the population level and in the cousin comparisons (Fig. 1, Supplementary Table S13). The distributions of specific indicators across the sum score categories are presented in Supplementary Fig. S1. The associations between one, two, three, and four or more household dysfunction indicators and the outcomes were on average 8% (range 4%–17%), 14% (4%–25%), 20% (11%–34%), and 18% (1%–34%) smaller in the cousin comparisons than in the population-level models, respectively (Supplementary Table S13). The largest attenuations were observed for offspring property crime arrest, followed by violent crime arrests and hospital-presenting substance use. In around half of the comparisons, the CIs from cousin comparisons and population-level models overlapped.

### Additional analyses

Excluding the individuals with outcomes before the start of follow-up did not have a major impact on the results (Supplementary Table S14 and Supplementary Figs S2 and S3). The factor analysis of the household dysfunction indicators (Supplementary Tables S15 and S16) pointed towards the exposures belonging to one general adversity domain, but with parental death and to some extent union dissolution as outliers.

## Discussion

In this study, we demonstrated the well-known associations between multiple indicators of childhood household dysfunction and multiple outcomes in emerging adulthood, and the dose–response relationship between the exposures and the outcomes [1–11]. Our approach allowed us to compare the magnitude of these different association in a well-characterized data with consistent measurement and no loss to follow-up over time. We also extended previous research by using cousin comparisons to assess unobserved confounding in these associations, an approach seldom used in previous research on childhood household dysfunction (for exceptions, see Refs. [32,34,40–42])

Based on previous studies [22, 30, 32, 34], we expected to observe familial confounding in all the associations, and, indeed, in nearly all the exposure-outcome associations, the estimate from the cousin comparison was lower than the estimate from the population-level model. On average, the estimates attenuated by about 12%, but the range of attenuation was wide, from null to 39%. In over half of the associations, the CIs from the population-level model and cousin comparison overlapped, indicating uncertainty in the difference between the estimates. This might partly relate to the loss of statistical power in the cousin comparisons as the overlap occurred less frequently with the most common household dysfunction indicators (social assistance and union dissolution).

Due to the intergenerational components of the exposures, we expected to find more familial confounding in the associations between similar parental and offspring variables than in the other exposure-outcome associations. Such a pattern did not clearly emerge from the data. Regarding the dose–response relationship, we expected to find more familial confounding in the associations between higher number of exposures and the outcomes, which was confirmed.

Across the single exposures and the sum score of the exposures, the largest attenuations between the population-level model and the cousin comparison were observed in the associations between the exposures and offspring violent crime, property crime and hospital-presenting substance use, which might relate to higher degree of heritability and genetic correlation between these outcomes [14, 43, 44]. The larger amount of unobserved familial confounding also led to less of an overlap between the CIs from the population-level models and the cousin comparisons. The difference between the population-level model and the cousin comparison was especially clear for property crime.

Of the specific exposures, the associations between parental prison sentence and the outcomes seemed to exhibit a larger amount of familial confounding, which is likely related to the very high prevalence of different psychiatric, personality, and substance use disorders as well as socioeconomic disadvantage among Finnish prisoners [45, 46]. Parental death seemed to differ from the other household dysfunction indicators, which was also observed in the factor analysis: the estimates from cousin comparisons were very close to estimates from population-level models for all outcomes, except offspring substance use.

### Methodological considerations

Although the cousin comparison addresses some of the problems of concordant exposures within nuclear families, it only accounts for approximately 12.5% of co-segregating genes and those environmental factors that are shared on the extended family level. Therefore, both genetic and social confounding in these associations is likely to remain. Second, we did not assess the shares of confounding attributable to environmental or genetic factors, which would require extending the cousin comparison to a children-of-siblings or children-of-twins [27–29] design. Third, due to the non-collapsibility of HRs [47] and inflation of measurement error in family-based designs [48], part of the attenuation observed between different models might be artificial. Fourth, register data only include relatively severe exposures and outcomes, and our results might not extend to associations between less severe exposures and outcomes. On the other hand, our results are based on objectively assessed conditions with long-term follow-up, little attrition, and no response biases.

Our observations highlight the need for prevention and intervention programmes at different societal levels. Since some of the associations between specific indicators and the outcomes were less confounded, preventing these experiences might decrease the risk of adverse outcomes at the population level [22]. Second, since most of the associations seemed to exhibit some level of unobserved confounding, and since some of the experiences might be hard to prevent, targeted support for children exposed to household dysfunction in early life are also needed to mitigate the negative consequences of these experiences [49]. Potential opportunities for evaluation and intervention may present themselves when parents are in contact with healthcare or other authorities, such as social services or the judicial system, but also in more routine settings, such as primary care [50]. Due to the broad range of both the exposures and outcomes, it seems evident that multidisciplinary intervention efforts are required.

## Supplementary Material

dyaf074_Supplementary_Data

## Data Availability

Finnish privacy laws and other data protection regulations prohibit publishing the underlying individual-level data used in this study. Researchers interested in replicating the work or doing similar analyses may seek access via Statistics Finland (https://stat.fi/tup/tutkijapalvelut/index_en.html) and Findata (https://findata.fi/en/permits/).
