# Supplementary material for: Childhood household dysfunction and psychiatric, criminal, and social outcomes in emerging adulthood. A cousin comparison study
Source: Int J Epidemiol. 2025 May 29;54(3):dyaf074. doi: 10.1093/ije/dyaf074 (PMC12122080; doi:10.1093/ije/dyaf074)
Supplement: dyaf074_Supplementary_Data [file dyaf074_supplementary_data.docx]

**Supplementary material to Childhood household dysfunction and psychiatric, criminal and social outcomes in emerging adulthood. A cousin comparison study**

[Supplementary table S1: Definitions of the childhood household dysfunction indicators 2](#_Toc191567048)

[Supplementary table S2: Definitions of the outcome variables 3](#_Toc191567049)

[Supplementary table S3: Definitions of the observed confounders 4](#_Toc191567050)

[Supplementary table S4: Number of events and proportion of individuals with an outcome by indicators of childhood household dysfunction 5](#_Toc191567051)

[Supplementary table S5: Distribution of the observed confounders and rates of outcomes (per 1000 person-years) across confounder categories 6](#_Toc191567052)

[Supplementary table S6: Number of events and proportion of individuals with an outcome by confounder categories 7](#_Toc191567053)

[Supplementary table S7: Distribution of the household dysfunction indicators and rates of outcomes (per 1000 person-years) across categories of the indicators in the cousin population (n=87 500) 8](#_Toc191567054)

[Supplementary table S8: Number of events and proportion of individuals with an outcome by indicators of household dysfunction in the cousin population (n=87 500) 9](#_Toc191567055)

[Supplementary table S9: Distribution of the observed confounders and rates of outcomes (per 1,000 person-years) across confounder categories in the cousin population (n=87 500) 10](#_Toc191567056)

[Supplementary table S10: Number of events and proportion of individuals with an outcome by confounder categories in the cousin population (n=87 500) 11](#_Toc191567057)

[Supplementary table S11: P-values from all the regression models, adjusted for multiple testing with the False Discovery Rate (FDR) method 12](#_Toc191567058)

[Supplementary table S12: Attenuation between population-level covariate-adjusted models and cousin comparisons and average attenuation across all exposures and outcomes. 16](#_Toc191567059)

[Supplementary table S13: Associations between the sum score of indicators of household dysfunction and psychiatric, criminal and social outcomes in the total population and in the cousin comparison, and percentage attenuated between the population model and the cousin comparison. 17](#_Toc191567060)

[Supplementary table S14: Number and proportion of individuals excluded from the total in the sensitivity analyses including only incident outcomes 18](#_Toc191567061)

[Supplementary table S15: Tetrachoric correlations between the childhood household dysfunction indicators in the total population (n=835 987) 19](#_Toc191567062)

[Supplementary table S16: Results from an exploratory factor analysis on a tetrachoric correlation matrix of the childhood household dysfunction indicators 19](#_Toc191567063)

[Supplementary figure S1: Proportions of the total sum of household dysfunction indicators accounted by each specific indicator across the categories of the sum score variable. 20](#_Toc191567064)

[Supplementary figure S2: Associations between single indicators of household dysfunction and psychiatric and criminal outcomes in emerging adulthood in crude and confounder-adjusted models in the total population and in confounder-adjusted model in the cousin comparison. Results from the main analysis and sensitivity analysis excluding prevalent outcomes. 21](#_Toc191567065)

[Supplementary figure S3: Associations between the sum score of indicators of household dysfunction and psychiatric and criminal outcomes in the total population and in the cousin comparison. Results from the main analysis and sensitivity analysis excluding prevalent outcomes. 22](#_Toc191567066)

Supplementary table S1: Definitions of the childhood household dysfunction indicators

| **Exposure**  (measured at child’s ages 0–14) | **Definition** | **Data source** | **Data availability** |
| --- | --- | --- | --- |
| Parental inpatient episode due to psychiatric disorders | At least one of the child's biological parents had an inpatient episode with at least one of the following diagnoses: schizophrenia (ICD9: 295, 297, 298; ICD10: F20–F29), mood disorder (ICD9: 2961–2698, 3004A, 3011D; ICD10: F30–F39), neurotic, stress-related and somatoform disorders [ICD9: 300 (excl. 3004A), 309; ICD10: F40–F48]. Primary and contributory diagnoses were included. Measured when the child was 0–14 years old. | Care Register for Health Care, Finnish Institute for Health and Welfare | 1970-2020 |
| Parental inpatient episode due to substance use | At least one of the child's biological parents had an inpatient episode related to alcohol (ICD9: 2650A, 291, 303, 3050, 3275, 4255, 5307A, 5353, 5710–5713, 5770–5770F, 5771C, 5771D, 980; ICD10: F10, E244, E52, G312, G4051, G621, G721, I426, K292, K70, K852, K860, R780, T51, X45, Y90, Y91, Z502, Z714, Z721) or drug use [ICD9: 0704, 0705, 292, 304, 3052–3059, 9650, 9658, 9659, 967, 969, 970 (excl. 9701A); ICD10: F11–F16, F18–F19, B171, B182, R781–R785, T36^a^, T40, X44 ^a^, Z503, Z715, Z722]. Primary and contributory diagnoses were included. Measured when the child was 0–14 years old. | Care Register for Health Care, Finnish Institute for Health and Welfare | 1970-2020 |
| Parental death | At least one of the child's biological parents died. Measured when the child was 0–14 years old. | Causes of Death, Statistics Finland | 1970-2020 |
| Parental prison sentence | At least one of the child's biological parents was sentenced to prison. Measured when the child was 0–14 years old. | Criminal Sentence Register | 1977-2020 |
| Parental union dissolution | Child's biological parents experienced a union dissolution either from a cohabiting union or a marriage. Measured when the child was 0–14 years old. | FOLK module for household unions, Statistics Finland | 1987-2020 |
| Parental social assistance | Either one of the child's biological parents received social assistance (>0€). Measured when the child was 0–14 years old. | FOLK module for income, Statistics Finland | 1987-2019 |
| ^a^Diagnoses T36 and X44 were used in combination with the Anatomical Therapeutic Chemical Classification system codes N02A, N05, N06, N07BC  ICD9/10: International Classification of Diseases version 9 or 10 (Finnish version) | | | |

Supplementary table S2: Definitions of the outcome variables

| **Outcome** | **Definition** | **Data source** | **Data availability** |
| --- | --- | --- | --- |
| Hospital-presenting psychiatric disorder | The child had an inpatient episode or a visit to specialized outpatient care with at least one of the following diagnoses: schizophrenia (ICD9: 295, 297, 298; ICD10: F20–F29), mood disorder (ICD9: 2961–2698, 3004A, 3011D; ICD10: F30–F39), neurotic, stress-related and somatoform disorders [ICD9: 300 (excl. 3004A), 309; ICD10: F40–F48] during follow-up. Primary and contributory diagnoses were included. | Care Register for Health Care, Finnish Institute for Health and Welfare | Inpatient data 1970-2020, outpatient data 1998-2020 |
| Hospital-presenting substance use | The child had an inpatient episode or a visit to specialized outpatient care with at least one of the following diagnoses: alcohol (ICD9: 2650A, 291, 303, 3050, 3275, 4255, 5307A, 5353, 5710–5713, 5770–5770F, 5771C, 5771D, 980; ICD10: F10, E244, E52, G312, G4051, G621, G721, I426, K292, K70, K852, K860, R780, T51, X45, Y90, Y91, Z502, Z714, Z721) or drug use [ICD9: 0704, 0705, 292, 304, 3052–3059, 9650, 9658, 9659, 967, 969, 970 (excl. 9701A); ICD10: F11–F16, F18–F19, B171, B182, R781–R785, T36^a^, T40, X44^a^, Z503, Z715, Z722] during follow-up. Primary and contributory diagnoses were included. | Care Register for Health Care, Finnish Institute for Health and Welfare | Inpatient data 1970-2020, outpatient data 1998-2020 |
| Psychotropic medication purchase | The child had purchased medication from any of the following ATC classes: antipsychotics (N05A), anxiolytics (N05B), hypnotics and sedatives (N05C), antidepressants (N06A), psychostimulants (N06C), psycholeptics and psychoanaleptics in combination (N06C) during follow-up. | Register of Prescription Medication Purchases, The Social Insurance Institution of Finland | 1995-2020 |
| Violent crime arrest | The child was suspected of any violent crime (offences against life and health as defined by Statistics Finland in 2015 classification) during follow-up. | Offences known to police, Statistics Finland | 1996-2020 |
| Property crime arrest | The child was suspected of any property crime (offences against property as defined by Statistics Finland in 2015 classification) during follow-up. | Offences known to police, Statistics Finland | 1996-2020 |
| Not in education, employment or training | The child was not in education, employment, training or military service during the last week of any year, during two consecutive years. | FOLK Basic module, Statistics Finland | 1987-2019 |
| ^a^Diagnoses T36 and X44 were used in combination with the Anatomical Therapeutic Chemical Classification system codes N02A, N05, N06, N07BC  ICD9/10: International Classification of Diseases version 9 or 10 (Finnish version) | | | |

| **Observed confounder** | **Definition** | **Data source** | **Data availability** |
| --- | --- | --- | --- |
| Birth cohort | Child’s year of birth, combined into three categories: 1987–1991, 1992–1996, 1997–2000 | Statistics Finland | Every individual in data |
| Sex | Latest observation of person’s sex (male and female). | FOLK Basic module, Statistics Finland | 1987–2020 |
| Region of residence | Region of residence at age 0 at NUTS2 level (Western Finland (incl. Åland), Helsinki-Uusimaa, Southern Finland, Northern and Eastern Finland) | FOLK Basic module, Statistics Finland | 1987–2020 |
| Parental education | Highest education of either biological mother or father at age 0. Categorized into no secondary education (ISCED 0–2), upper secondary education (ISCED 3–4), lower tertiary education (ISCED 5–6), higher tertiary education (ISCED 7–8). | FOLK Basic module, Statistics Finland | 1987–2020 |
| Two-parent family | A binary indicator measuring if the child resided in a two-parent family at age 0. | FOLK Basic module, Statistics Finland | 1987–2020 |
| Maternal age at birth | Calculated based on mother’s and child’s birth years and categorized into < 25, 25–34, >35. | Statistics Finland | Every individual in data |
| Birth order | Order of birth within each mother’s full fertility history (calculation includes children born to these mothers outside the included birth cohorts 1987–2000). | Statistics Finland | Every individual in data |
| ISCED: International Standard Classification of Education  NUTS2: Nomenclature of territorial units for statistics (EU classification) | | | |

Supplementary table S3: Definitions of the observed confounders

Supplementary table S4: Number of events and proportion of individuals with an outcome by indicators of childhood household dysfunction

|  |  | Offspring outcomes | | | | | |
| --- | --- | --- | --- | --- | --- | --- | --- |
| Household dysfunction indicator (measured from parents at child’s ages 0–14) | Total | Hospital-presenting psychiatric disorder | Hospital-presenting substance use | Psychotropic medication purchase | Two-year NEET | Violent crime arrest | Property crime arrest |
|  | n | Events [%] | Events [%] | Events [%] | Events [%] | Events [%] | Events [%] |
| Parental psychiatric hospitalisation |  |  |  |  |  |  |  |
| No | 784 405 | 124 054 [16] | 37 459 [5] | 194 320 [25] | 175 634 [22] | 47 202 [6] | 77 281 [10] |
| Yes | 51 582 | 15 140 [29] | 5024 [10] | 20 230 [39] | 17 043 [33] | 5573 [11] | 8938 [17] |
| Parental substance-attributable hospitalisation |  |  |  |  |  |  |  |
| No | 793 904 | 127 096 [16] | 37 231 [5] | 197 842 [25] | 176 648 [22] | 46 536 [6] | 76 755 [10] |
| Yes | 42 083 | 12 098 [29] | 5252 [12] | 16 708 [40] | 16 029 [38] | 6239 [15] | 9464 [22] |
| Parental death |  |  |  |  |  |  |  |
| No | 810 842 | 132 899 [16] | 40 120 [5] | 205 861 [25] | 184 382 [23] | 50 017 [6] | 81 889 [10] |
| Yes | 25 145 | 6295 [25] | 2363 [9] | 8689 [35] | 8295 [33] | 2758 [11] | 4330 [17] |
| Parental prison sentence |  |  |  |  |  |  |  |
| No | 821 564 | 134 786 [16] | 40 031 [5] | 208 266 [25] | 185 791 [23] | 49 316 [6] | 81 257 [10] |
| Yes | 14 423 | 4408 [31] | 2452 [17] | 6284 [44] | 6886 [48] | 3459 [24] | 4962 [34] |
| Parental union dissolution |  |  |  |  |  |  |  |
| No | 581 578 | 82 313 [14] | 22 633 [4] | 132 933 [23] | 118 345 [20] | 28 131 [5] | 45 978 [8] |
| Yes | 254 409 | 56 881 [22] | 19 850 [8] | 81 617 [32] | 74 332 [29] | 24 644 [10] | 40 241 [16] |
| Parental social assistance |  |  |  |  |  |  |  |
| No | 549 403 | 72 555 [13] | 18 486 [3] | 118 952 [22] | 94 858 [17] | 20 916 [4] | 36 257 [7] |
| Yes | 286 584 | 66 639 [23] | 23 997 [8] | 95 598 [33] | 97 819 [34] | 31 859 [11] | 49 962 [17] |
| Total number of indicators |  |  |  |  |  |  |  |
| 0 | 431 740 | 52 219 [12] | 12 860 [3] | 88 183 [20] | 71 966 [17] | 14 954 [3] | 25 586 [6] |
| 1 | 214 391 | 37 966 [18] | 11 334 [5] | 57 757 [27] | 53 682 [25] | 14 279 [7] | 23 407 [11] |
| 2 | 134 481 | 31 924 [24] | 11 160 [8] | 45 613 [34] | 44 977 [33] | 14 870 [11] | 23 931 [18] |
| 3 | 35 254 | 10 394 [29] | 4064 [12] | 14 083 [40] | 13 548 [38] | 5118 [15] | 7956 [23] |
| 4 or more | 20 121 | 6691 [33] | 3065 [15] | 8914 [44] | 8504 [42] | 3554 [18] | 5339 [27] |
| Total | 835 987 | 139 194 [17] | 42 483 [5] | 214 550 [26] | 192 677 [23] | 52 775 [6] | 86 219 [10] |
| NEET: Not in education, employment, or training | | | | | | | |

Supplementary table S5: Distribution of the observed confounders and rates of outcomes (per 1000 person-years) across confounder categories

|  |  | Offspring outcomes | | | | | |
| --- | --- | --- | --- | --- | --- | --- | --- |
| Observed confounder (measured at age 0) | Total | Hospital-presenting psychiatric disorder psychiatric | Hospital-presenting substance use | Psychotropic medication purchases | Two-year NEET | Violent crime arrest | Property crime arrest |
| Birth cohort | n [%] | Rate [95% CI] | Rate [95% CI] | Rate [95% CI] | Rate [95% CI] | Rate [95% CI] | Rate [95% CI] |
| 1987–1991 | 306 198 [37] | 11.7 [11.6,11.8] | 3.5 [3.5,3.6] | 23.3 [23.3,23.5] | 22.8 [22.7,23.0] | 5.3 [5.2,5.4] | 8.2 [8.1,8.3] |
| 1992–1996 | 308 474 [37] | 15.8 [15.7,15.9] | 4.6 [4.5,4.6] | 26.6 [26.4,26.8] | 28.3 [28.1,28.5] | 5.4 [5.4,5.5] | 9.8 [9.7,9.9] |
| 1997–2000 | 221 315 [26] | 21.4 [21.2,21.7] | 5.4 [5.3,5.5] | 31.5 [31.2,31.8] | 22.1 [21.8,22.3] | 4.9 [4.8,5.0] | 9.7 [9.5,9.9] |
| Child's sex |  |  |  |  |  |  |  |
| Men | 427 133 [51] | 10.6 [10.5,10.7] | 4.7 [4.7,4.8] | 20.1 [20.0,20.2] | 27.9 [27.7,28.1] | 7.8 [7.7,7.9] | 12.2 [12.1,12.3] |
| Women | 408 854 [49] | 19.3 [19.2,19.5] | 3.6 [3.6,3.7] | 32.1 [32.0,32.3] | 21.3 [21.2,21.5] | 2.8 [2.7,2.8] | 5.8 [5.7,5.9] |
| Region of residence at birth |  |  |  |  |  |  |  |
| Helsinki and Uusimaa | 227 644 [27] | 17.1 [17.0,17.3] | 4.3 [4.2,4.3] | 27.1 [26.9,27.3] | 20.8 [20.6,21.0] | 5.1 [5.0,5.2] | 9.8 [9.7,9.9] |
| Southern Finland | 171 540 [21] | 14.6 [14.4,14.7] | 4.5 [4.4,4.6] | 25.4 [25.2,25.7] | 25.6 [25.3,25.8] | 5.3 [5.2,5.4] | 9.1 [9.0,9.3] |
| Western Finland | 213 242 [26] | 13.1 [12.9,13.3] | 4.0 [3.9,4.0] | 25.1 [24.9,25.4] | 25.0 [24.8,25.2] | 5.5 [5.5,5.7] | 8.9 [8.8,9.0] |
| Northern and Eastern Finland | 223 561 [27] | 14.0 [13.8,14.2] | 4.1 [4.0,4.2] | 25.3 [25.1,25.5] | 27.5 [27.2,27.7] | 5.2 [5.1,5.3] | 8.2 [8.1,8.3] |
| Parental education at birth |  |  |  |  |  |  |  |
| No secondary | 63 267 [8] | 21.1 [20.7,21.5] | 8.0 [7.7,8.2] | 35.4 [34.9,35.9] | 45.9 [45.3,46.5] | 12.5 [12.2,12.7] | 20.3 [19.9,20.7] |
| Upper secondary | 369 454 [44] | 15.6 [15.5,15.8] | 4.7 [4.7,4.8] | 27.2 [27.0,27.3] | 29.8 [29.6,30.0] | 6.6 [6.5,6.7] | 10.8 [10.7,10.9] |
| Lower tertiary | 291 400 [35] | 12.9 [12.7,13.0] | 3.2 [3.2,3.3] | 22.8 [22.7,23.0] | 18.3 [18.2,18.5] | 3.4 [3.4,3.5] | 6.2 [6.1,6.3] |
| Higher tertiary | 111 866 [13] | 12.7 [12.5,12.9] | 2.6 [2.5,2.7] | 23.4 [23.1,23.7] | 13.3 [13.1,13.6] | 1.8 [1.7,1.9] | 4.2 [4.1,4.4] |
| Two-parent family at birth |  |  |  |  |  |  |  |
| No | 58 105 [7] | 21.5 [21.1,21.9] | 7.4 [7.2,7.7] | 34.8 [34.3,35.3] | 36.3 [35.8,36.9] | 10.0 [9.8,10.3] | 16.9 [16.6,17.3] |
| Yes | 777 882 [93] | 14.2 [14.2,14.3] | 4.0 [3.9,4] | 25.2 [25.0,25.3] | 23.9 [23.7,24.0] | 5.0 [4.9,5.0] | 8.5 [8.4,8.5] |
| Maternal age at childbirth |  |  |  |  |  |  |  |
| Under 25 | 145 741 [17] | 18.0 [17.7,18.2] | 6.3 [6.1,6.4] | 30.8 [30.5,31.1] | 32.9 [32.6,33.2] | 9.2 [9.0,9.4] | 15.0 [14.8,15.2] |
| 25–34 | 546 440 [65] | 13.7 [13.6,13.8] | 3.8 [3.7,3.8] | 24.4 [24.2,24.5] | 22.7 [22.5,22.8] | 4.7 [4.6,4.7] | 8.0 [7.9,8.1] |
| 35 or older | 143 806 [17] | 15.3 [15.1,15.5] | 3.6 [3.5,3.7] | 26.2 [25.9,26.4] | 24.1 [23.8,24.4] | 3.8 [3.7,3.9] | 7.0 [6.9,7.1] |
| Birth order |  |  |  |  |  |  |  |
| First born | 328 484 [39] | 15.0 [14.9,15.2] | 4.0 [4.0,4.1] | 26.3 [26.1,26.5] | 22.6 [22.5,22.8] | 5.0 [4.9,5.1] | 8.8 [8.7,8.9] |
| Second born | 287 767 [34] | 14.0 [13.9,14.1] | 4.2 [4.1,4.3] | 24.9 [24.7,25.1] | 24.2 [24.0,24.4] | 5.3 [5.2,5.4] | 8.8 [8.7,8.9] |
| Third born | 142 061 [17] | 14.6 [14.4,14.8] | 4.3 [4.2,4.4] | 25.7 [25.5,26.0] | 26.7 [26.4,26.9] | 5.5 [5.4,5.6] | 9.2 [9.0,9.3] |
| Fourth born or later | 77 675 [9] | 16.1 [15.9,16.4] | 4.6 [4.5,4.8] | 26.9 [26.5,27.3] | 31.6 [31.2,32.1] | 6.2 [6.0,6.3] | 10.0 [9.8,10.2] |
| NEET: Not in education, employment or training | | | | | | | |

Supplementary table S6: Number of events and proportion of individuals with an outcome by confounder categories

|  |  | Offspring outcomes | | | | | |
| --- | --- | --- | --- | --- | --- | --- | --- |
| Observed confounder (measured at age 0) | Total | Hospital-presenting psychiatric disorder | Hospital-presenting substance use | Psychotropic medication purchase | Two-year NEET | Violent crime arrest | Property crime arrest |
|  | n | Events [%] | Events [%] | Events [%] | Events [%] | Events [%] | Events [%] |
| Birth cohort |  |  |  |  |  |  |  |
| 1987–1991 | 306 198 | 54 067 [18] | 17 389 [6] | 94 996 [31] | 88 767 [29] | 25 563 [8] | 37 924 [12] |
| 1992–1996 | 308 474 | 52 603 [17] | 16 307 [5] | 78 597 [25] | 77 859 [25] | 19 208 [6] | 32 993 [11] |
| 1997–2000 | 221 315 | 32 524 [15] | 8787 [4] | 40 957 [19] | 26 051 [12] | 8004 [4] | 15 302 [7] |
| Child's sex |  |  |  |  |  |  |  |
| Men | 427 133 | 52 769 [12] | 24 417 [6] | 88 497 [21] | 110 060 [26] | 38 939 [9] | 58 278 [14] |
| Women | 408 854 | 86 425 [21] | 18 066 [4] | 126 053 [31] | 82 617 [20] | 13 836 [3] | 27 941 [7] |
| Region of residence at birth |  |  |  |  |  |  |  |
| Helsinki and Uusimaa | 227 644 | 42 603 [19] | 11 535 [5] | 59 978 [26] | 44 288 [19] | 13 575 [6] | 24 923 [11] |
| Southern Finland | 171 540 | 28 422 [17] | 9428 [5] | 43 632 [25] | 40 961 [24] | 10 952 [6] | 17 985 [10] |
| Western Finland | 213 242 | 31 981 [15] | 10 259 [5] | 53 583 [25] | 49 850 [23] | 14 097 [7] | 21 774 [10] |
| Northern and Eastern Finland | 223 561 | 36 188 [16] | 11 261 [5] | 57 357 [26] | 57 578 [26] | 14 151 [6] | 21 537 [10] |
| Parental education at birth |  |  |  |  |  |  |  |
| No secondary | 63 267 | 14 943 [24] | 6166 [10] | 21 767 [34] | 25 133 [40] | 9195 [15] | 13 821 [22] |
| Upper secondary | 369 454 | 66 517 [18] | 21 728 [6] | 101 473 [27] | 102 899 [28] | 29 370 [8] | 46 184 [13] |
| Lower tertiary | 291 400 | 42 210 [14] | 11 229 [4] | 66 194 [23] | 50 655 [17] | 11 836 [4] | 20 817 [7] |
| Higher tertiary | 111 866 | 15 524 [14] | 3360 [3] | 25 116 [22] | 13 990 [13] | 2374 [2] | 5397 [5] |
| Two-parent family at birth |  |  |  |  |  |  |  |
| No | 58 105 | 12 999 [22] | 4930 [8] | 18 349 [32] | 17 865 [31] | 6453 [11] | 10 166 [17] |
| Yes | 777 882 | 126 195 [16] | 37 553 [5] | 196 201 [25] | 174 812 [22] | 46 322 [6] | 76 053 [10] |
| Maternal age at childbirth |  |  |  |  |  |  |  |
| Under 25 | 145 741 | 29 550 [20] | 11 123 [8] | 44 266 [30] | 43 811 [30] | 15 812 [11] | 24 163 [17] |
| 25–34 | 546 440 | 85 594 [16] | 25 309 [5] | 134 223 [25] | 117 567 [22] | 30 601 [6] | 50 708 [9] |
| 35 or older | 143 806 | 24 050 [17] | 6051 [4] | 36 061 [25] | 31 299 [22] | 6362 [4] | 11 348 [8] |
| Birth order |  |  |  |  |  |  |  |
| First born | 328 484 | 55 734 [17] | 16 068 [5] | 85 680 [26] | 70 241 [21] | 19 641 [6] | 33 246 [10] |
| Second born | 287 767 | 46 022 [16] | 14 692 [5] | 72 056 [25] | 65 562 [23] | 18 253 [6] | 29 305 [10] |
| Third born | 142 061 | 23 630 [17] | 7479 [5] | 36 571 [26] | 35 232 [25] | 9323 [7] | 14 993 [11] |
| Fourth born or later | 77 675 | 13 808 [18] | 4244 [5] | 20 243 [26] | 21 642 [28] | 5558 [7] | 8675 [11] |
| NEET: Not in education, employment or training | | | | | | | |

Supplementary table S7: Distribution of the household dysfunction indicators and rates of outcomes (per 1000 person-years) across categories of the indicators in the cousin population (n=87 500)

|  |  | Offspring outcomes | | | | | |
| --- | --- | --- | --- | --- | --- | --- | --- |
| Household dysfunction indicators (measured from parents at child’s ages 0–14) | Total | Hospital-presenting psychiatric disorder | Hospital-presenting substance use | Psychotropic medication purchases | Two-year NEET | Violent crime arrest | Property crime arrest |
| Parental psychiatric hospitalisation | n [%] | Rate [95% CI] | Rate [95% CI] | Rate [95% CI] | Rate [95% CI] | Rate [95% CI] | Rate [95% CI] |
| No | 82 429 [94] | 13.4 [13.2,13.7] | 3.6 [3.5,3.7] | 24.4 [24.0,24.7] | 21.5 [21.2,21.8] | 4.6 [4.5,4.8] | 8.0 [7.9,8.2] |
| Yes | 5071 [6] | 27.6 [26.2,29.1] | 7.3 [6.7,8.1] | 42.5 [40.6,44.4] | 35.9 [34.3,37.6] | 8.6 [7.9,9.4] | 15.1 [14.0,16.2] |
| Parental substance-attributable hospitalisation |  |  |  |  |  |  |  |
| No | 83 549 [95] | 13.7 [13.4,13.9] | 3.5 [3.4,3.6] | 24.6 [24.3,25.0] | 21.4 [21.1,21.7] | 4.5 [4.4,4.6] | 7.9 [7.7,8.1] |
| Yes | 3951 [5] | 25.8 [24.2,27.4] | 9.5 [8.7,10.4] | 41.0 [39.0,43.2] | 42.7 [40.6,44.8] | 12.1 [11.1,13.1] | 20.5 [19.1,22.0] |
| Parental death |  |  |  |  |  |  |  |
| No | 85 455 [98] | 14.0 [13.7,14.2] | 3.7 [3.6,3.8] | 25.0 [24.7,25.4] | 22.0 [21.7,22.4] | 4.7 [4.6,4.9] | 8.2 [8.1,8.4] |
| Yes | 2045 [2] | 23.4 [21.4,25.6] | 7.6 [6.6,8.8] | 37.1 [34.5,39.9] | 34.4 [31.9,37.1] | 9.1 [7.9,10.4] | 16.9 [15.3,18.8] |
| Parental prison sentence |  |  |  |  |  |  |  |
| No | 85 906 [98] | 13.9 [13.7,14.1] | 3.6 [3.5,3.8] | 24.9 [24.6,25.3] | 21.7 [21.4,22.1] | 4.6 [4.4,4.7] | 8.0 [7.9,8.2] |
| Yes | 1594 [2] | 29.6 [27.1,32.4] | 12.3 [10.8,14.0] | 47.3 [44.0,50.9] | 57.4 [53.6,61.5] | 20.1 [18.0,22.4] | 33.1 [30.1,36.5] |
| Parental union dissolution |  |  |  |  |  |  |  |
| No | 60 693 [69] | 11.8 [11.5,12.0] | 2.9 [2.8,3.0] | 21.9 [21.5,22.3] | 19.1 [18.7,19.4] | 3.7 [3.5,3.8] | 6.5 [6.3,6.7] |
| Yes | 26 807 [31] | 20.0 [19.5,20.5] | 5.8 [5.6,6.1] | 33.6 [32.9,34.3] | 30.1 [29.4,30.8] | 7.6 [7.3,7.9] | 13.1 [12.7,13.6] |
| Parental social assistance |  |  |  |  |  |  |  |
| No | 57 256 [65] | 11.1 [10.9,11.4] | 2.5 [2.4,2.7] | 20.9 [20.5,21.3] | 16.0 [15.7,16.4] | 2.9 [2.8,3] | 5.3 [5.1,5.5] |
| Yes | 30 244 [35] | 20.1 [19.6,20.6] | 6.1 [5.9,6.4] | 33.9 [33.3,34.6] | 34.8 [34.1,35.5] | 8.5 [8.2,8.9] | 14.6 [14.2,15.0] |
| Total number of parental indicators |  |  |  |  |  |  |  |
| 0 | 45 032 [51] | 10.0 [9.7,10.3] | 2.2 [2.1,2.3] | 19.4 [19.0,19.8] | 15.2 [14.9,15.6] | 2.6 [2.5,2.8] | 4.8 [4.6,5.0] |
| 1 | 22 914 [26] | 15.2 [14.8,15.7] | 4.2 [4.0,4.5] | 26.7 [26.0,27.4] | 24.5 [23.9,25.2] | 5.0 [4.8,5.3] | 9.0 [8.6,9.4] |
| 2 | 14 210 [16] | 21.2 [20.5,22.0] | 6.1 [5.7,6.4] | 36.1 [35.1,37.1] | 34.5 [33.6,35.5] | 8.7 [8.3,9.2] | 15.0 [14.4,15.7] |
| 3 | 3422 [4] | 26.2 [24.6,28.0] | 8.5 [7.7,9.5] | 41.6 [39.4,43.9] | 41.9 [39.7,44.1] | 11.9 [10.8,13.0] | 19.4 [18.0,21.0] |
| 4 or more | 1922 [2] | 32.9 [30.3,35.6] | 11.9 [10.6,13.4] | 49.8 [46.5,53.4] | 50.6 [47.5,54.0] | 14.7 [13.2,16.5] | 26.2 [23.9,28.7] |
| NEET: Not in education, employment or training | | | | | | | |

Supplementary table S8: Number of events and proportion of individuals with an outcome by indicators of household dysfunction in the cousin population (n=87 500)

|  |  | Offspring outcomes | | | | | |
| --- | --- | --- | --- | --- | --- | --- | --- |
| Household dysfunction indicators (measured from parents at child’s ages 0–14) | Total | Hospital-presenting psychiatric disorder | Hospital-presenting substance use | Psychotropic medication purchase | Two-year NEET | Violent crime arrest | Property crime arrest |
| Parental psychiatric hospitalisation | n [%] | Events [%] | Events [%] | Events [%] | Events [%] | Events [%] | Events [%] |
| No | 82 429 [94] | 12 855 [16] | 3666 [4] | 20 546 [25] | 17 221 [21] | 4647 [6] | 7796 [9] |
| Yes | 5071 [6] | 1471 [29] | 451 [9] | 1979 [39] | 1660 [33] | 517 [10] | 849 [17] |
| Parental substance-attributable hospitalisation |  |  |  |  |  |  |  |
| No | 83 549 [95] | 13 212 [16] | 3655 [4] | 20 986 [25] | 17 363 [21] | 4600 [6] | 7765 [9] |
| Yes | 3951 [5] | 1114 [28] | 462 [12] | 1539 [39] | 1518 [38] | 564 [14] | 880 [22] |
| Parental death |  |  |  |  |  |  |  |
| No | 85 455 [98] | 13 804 [16] | 3928 [5] | 21 796 [26] | 18 228 [21] | 4942 [6] | 8261 [10] |
| Yes | 2045 [2] | 522 [26] | 189 [9] | 729 [36] | 653 [32] | 222 [11] | 384 [19] |
| Parental prison sentence |  |  |  |  |  |  |  |
| No | 85 906 [98] | 13 809 [16] | 3874 [5] | 21 811 [25] | 18 109 [21] | 4803 [6] | 8116 [9] |
| Yes | 1594 [2] | 517 [32] | 243 [15] | 714 [45] | 772 [48] | 361 [23] | 529 [33] |
| Parental union dissolution |  |  |  |  |  |  |  |
| No | 60 693 [69] | 8393 [14] | 2205 [4] | 13 803 [23] | 11 385 [19] | 2731 [4] | 4698 [8] |
| Yes | 26 807 [31] | 5933 [22] | 1912 [7] | 8722 [33] | 7496 [28] | 2433 [9] | 3947 [15] |
| Parental social assistance |  |  |  |  |  |  |  |
| No | 57 256 [65] | 7400 [13] | 1793 [3] | 12 332 [22] | 9036 [16] | 2023 [4] | 3620 [6] |
| Yes | 30 244 [35] | 6926 [23] | 2324 [8] | 10 193 [34] | 9845 [33] | 3141 [10] | 5025 [17] |
| Total number of parental indicators |  |  |  |  |  |  |  |
| 0 | 45 032 [51] | 5306 [12] | 1219 [3] | 9135 [20] | 6807 [15] | 1452 [3] | 2596 [6] |
| 1 | 22 914 [26] | 4002 [17] | 1189 [5] | 6181 [27] | 5379 [23] | 1406 [6] | 2399 [10] |
| 2 | 14 210 [16] | 3383 [24] | 1071 [8] | 4990 [35] | 4559 [32] | 1497 [11] | 2401 [17] |
| 3 | 3422 [4] | 982 [29] | 362 [11] | 1358 [40] | 1299 [38] | 483 [14] | 727 [21] |
| 4 or more | 1922 [2] | 653 [34] | 276 [14] | 861 [45] | 837 [44] | 326 [17] | 522 [28] |
| Total | 87 500 | 14 326 [16] | 4117 [5] | 22 525 [26] | 18 881 [22] | 5164 [6] | 8645 [10] |
| NEET: Not in education, employment or training | | | | | | | |

Supplementary table S9: Distribution of the observed confounders and rates of outcomes (per 1,000 person-years) across confounder categories in the cousin population (n=87 500)

|  |  | Offspring outcomes | | | | | |
| --- | --- | --- | --- | --- | --- | --- | --- |
| Observed confounder (measured at age 0) | Total | Hospital-presenting psychiatric disorder | Hospital-presenting substance use | Psychotropic medication purchases | Two-year NEET | Violent crime arrest | Property crime arrest |
| Birth cohort | n [%] | Rate [95% CI] | Rate [95% CI] | Rate [95% CI] | Rate [95% CI] | Rate [95% CI] | Rate [95% CI] |
| 1987–1991 | 33 717 [39] | 11.7 [11.4,12.0] | 3.4 [3.3,3.6] | 23.1 [22.6,23.5] | 21.2 [20.8,21.6] | 5.1 [4.9,5.3] | 8.2 [7.9,8.4] |
| 1992–1996 | 33 378 [38] | 15.1 [14.7,15.5] | 4.0 [3.8,4.2] | 26.2 [25.7,26.8] | 24.9 [24.4,25.5] | 4.8 [4.6,5.0] | 8.8 [8.5,9.1] |
| 1997–2000 | 20 405 [23] | 20.8 [20.1,21.6] | 4.7 [4.3,5.0] | 31.2 [30.1,32.2] | 19.5 [18.7,20.3] | 4.2 [3.9,4.5] | 8.4 [7.9,8.9] |
| Child's sex |  |  |  |  |  |  |  |
| Men | 44 685 [51] | 10.2 [9.9,10.5] | 4.3 [4.2,4.5] | 19.5 [19.1,19.9] | 25.5 [25.1,26.0] | 7.3 [7.0,7.5] | 11.4 [11.1,11.7] |
| Women | 42 815 [49] | 18.6 [18.2,19.0] | 3.2 [3.1,3.4] | 31.8 [31.3,32.4] | 19.0 [18.6,19.5] | 2.4 [2.3,2.5] | 5.5 [5.3,5.7] |
| Region of residence at birth |  |  |  |  |  |  |  |
| Helsinki and Uusimaa | 23 656 [27] | 16.6 [16.1,17.1] | 3.7 [3.5,4.0] | 26.8 [26.1,27.4] | 18.5 [17.9,19.0] | 4.6 [4.4,4.9] | 8.9 [8.6,9.3] |
| Southern Finland | 17 522 [20] | 13.8 [13.2,14.3] | 4.0 [3.8,4.3] | 24.8 [24.0,25.5] | 23.0 [22.3,23.7] | 4.8 [4.5,5.1] | 8.5 [8.1,8.9] |
| Western Finland | 21 815 [25] | 12.8 [12.4,13.3] | 3.7 [3.5,3.9] | 24.5 [23.8,25.1] | 22.2 [21.5,22.8] | 5.1 [4.8,5.4] | 8.4 [8.0,8.7] |
| Northern and Eastern Finland | 24 507 [28] | 13.4 [13.0,13.9] | 3.8 [3.6,4.0] | 25.1 [24.5,25.7] | 25.7 [25.1,26.3] | 4.9 [4.6,5.1] | 8.0 [7.6,8.3] |
| Parental education at birth |  |  |  |  |  |  |  |
| No secondary | 5285 [6] | 22.2 [21,23.5] | 8.0 [7.4,8.7] | 37.4 [35.8,39.2] | 46.9 [45.0,48.9] | 13.1 [12.2,14.1] | 22.4 [21.1,23.8] |
| Upper secondary | 38 961 [45] | 14.7 [14.4,15.1] | 4.2 [4.1,4.4] | 26.5 [26.0,27.0] | 26.8 [26.3,27.3] | 6.0 [5.8,6.2] | 10.0 [9.8,10.4] |
| Lower tertiary | 31 730 [36] | 12.7 [12.3,13.1] | 3.0 [2.8,3.2] | 22.5 [21.9,23.0] | 16.6 [16.2,17.1] | 3.2 [3.0,3.4] | 5.8 [5.5,6.0] |
| Higher tertiary | 11 524 [13] | 12.4 [11.8,13.1] | 2.4 [2.1,2.7] | 23.4 [22.5,24.3] | 12.2 [11.6,12.8] | 1.6 [1.4,1.8] | 4.1 [3.8,4.5] |
| Two-parent family at birth |  |  |  |  |  |  |  |
| No | 7688 [9] | 18.7 [17.8,19.7] | 6.1 [5.6,6.6] | 31.8 [30.5,33.1] | 31.8 [30.6,33.2] | 8.6 [8.0,9.3] | 14.7 [13.8,15.5] |
| Yes | 79 812 [91] | 13.8 [13.5,14] | 3.6 [3.5,3.7] | 24.7 [24.4,25.1] | 21.4 [21.1,21.8] | 4.5 [4.4,4.6] | 7.9 [7.7,8.1] |
| Maternal age at childbirth |  |  |  |  |  |  |  |
| Under 25 | 27 295 [31] | 16.0 [15.6,16.5] | 5.2 [5.0,5.5] | 28.9 [28.2,29.5] | 29.5 [28.9,30.2] | 7.8 [7.5,8.1] | 12.8 [12.4,13.2] |
| 25–34 | 55 047 [63] | 13.1 [12.8,13.3] | 3.1 [3.0,3.2] | 23.5 [23.1,23.9] | 18.9 [18.5,19.2] | 3.5 [3.4,3.6] | 6.4 [6.3,6.7] |
| 35 or older | 5158 [6] | 16.3 [15.2,17.5] | 3.3 [2.8,3.8] | 25.9 [24.4,27.4] | 20.3 [19.0,21.6] | 2.9 [2.5,3.4] | 6.1 [5.5,6.9] |
| NEET: Not in education, employment or training | | | | | | | |

Supplementary table S10: Number of events and proportion of individuals with an outcome by confounder categories in the cousin population (n=87 500)

|  |  | Offspring outcomes | | | | | |
| --- | --- | --- | --- | --- | --- | --- | --- |
| Observed confounder (measured at age 0) | Total | Hospital-presenting psychiatric disorder | Hospital-presenting substance use | Psychotropic medication purchase | Two-year NEET | Violent crime arrest | Property crime arrest |
| Birth cohort | n | Events [%] | Events [%] | Events [%] | Events [%] | Events [%] | Events [%] |
| 1987–1991 | 33 717 | 5910 [18] | 1852 [5] | 10 334 [31] | 9167 [27] | 2681 [8] | 4143 [12] |
| 1992–1996 | 33 378 | 5475 [16] | 1558 [5] | 8423 [25] | 7563 [23] | 1850 [6] | 3265 [10] |
| 1997–2000 | 20 405 | 2941 [14] | 707 [3] | 3768 [18] | 2151 [11] | 633 [3] | 1237 [6] |
| Child's sex |  |  |  |  |  |  |  |
| Men | 44 685 | 5412 [12] | 2394 [5] | 9183 [21] | 10 875 [24] | 3884 [9] | 5821 [13] |
| Women | 42 815 | 8914 [21] | 1723 [4] | 13 342 [31] | 8006 [19] | 1280 [3] | 2824 [7] |
| Region of residence at birth |  |  |  |  |  |  |  |
| Helsinki and Uusimaa | 23 656 | 4389 [19] | 1074 [5] | 6297 [27] | 4232 [18] | 1312 [6] | 2429 [10] |
| Etelä-Suomi | 17 522 | 2802 [16] | 876 [5] | 4432 [25] | 3891 [22] | 1026 [6] | 1756 [10] |
| Länsi-Suomi | 21 815 | 3259 [15] | 991 [5] | 5432 [25] | 4663 [21] | 1347 [6] | 2131 [10] |
| Pohjois- ja Itä-Suomi | 24 507 | 3876 [16] | 1176 [5] | 6364 [26] | 6095 [25] | 1479 [6] | 2329 [10] |
| Parental education at birth |  |  |  |  |  |  |  |
| No secondary | 5285 | 1340 [25] | 534 [10] | 1952 [37] | 2203 [42] | 825 [16] | 1287 [24] |
| Upper secondary | 38 961 | 6812 [17] | 2104 [5] | 10763 [28] | 10 218 [26] | 2901 [7] | 4660 [12] |
| Lower tertiary | 31 730 | 4595 [14] | 1156 [4] | 7193 [23] | 5123 [16] | 1221 [4] | 2150 [7] |
| Higher tertiary | 11 524 | 1579 [14] | 323 [3] | 2617 [23] | 1337 [12] | 217 [2] | 548 [5] |
| Two-parent family at birth |  |  |  |  |  |  |  |
| No | 7688 | 1595 [21] | 564 [7] | 2372 [31] | 2238 [29] | 777 [10] | 1240 [16] |
| Yes | 79 812 | 12 731 [16] | 3553 [4] | 20 153 [25] | 16 643 [21] | 4387 [5] | 7405 [9] |
| Maternal age at childbirth |  |  |  |  |  |  |  |
| Under 25 | 27 295 | 5235 [19] | 1836 [7] | 8240 [30] | 7903 [29] | 2660 [10] | 4127 [15] |
| 25–34 | 55 047 | 8280 [15] | 2106 [4] | 13 157 [24] | 10 141 [18] | 2349 [4] | 4199 [8] |
| 35 or older | 5158 | 811 [16] | 175 [3] | 1128 [22] | 837 [16] | 155 [3] | 319 [6] |
| NEET: Not in education, employment or training | | | | | | | |

Supplementary table S11: P-values from all the regression models, adjusted for multiple testing with the False Discovery Rate (FDR) method

| Indicator of household dysfunction | Offspring outcome | Model | FDR-adjusted p-value |
| --- | --- | --- | --- |
| Parental psychiatric hospitalisation | Hospital-presenting psychiatric disorder | Crude model, population | 0.00000 |
| Parental psychiatric hospitalisation | Hospital-presenting substance use | Crude model, population | 0.00000 |
| Parental psychiatric hospitalisation | Psychotropic medication purchase | Crude model, population | 0.00000 |
| Parental psychiatric hospitalisation | Two-year NEET | Crude model, population | 0.00000 |
| Parental psychiatric hospitalisation | Violent crime arrest | Crude model, population | 0.00000 |
| Parental psychiatric hospitalisation | Property crime arrest | Crude model, population | 0.00000 |
| Parental psychiatric hospitalisation | Hospital-presenting psychiatric disorder | Adjusted model, population | 0.00000 |
| Parental psychiatric hospitalisation | Hospital-presenting substance use | Adjusted model, population | 0.00000 |
| Parental psychiatric hospitalisation | Psychotropic medication purchase | Adjusted model, population | 0.00000 |
| Parental psychiatric hospitalisation | Two-year NEET | Adjusted model, population | 0.00000 |
| Parental psychiatric hospitalisation | Violent crime arrest | Adjusted model, population | 0.00000 |
| Parental psychiatric hospitalisation | Property crime arrest | Adjusted model, population | 0.00000 |
| Parental psychiatric hospitalisation | Hospital-presenting psychiatric disorder | Cousin comparison | 0.00000 |
| Parental psychiatric hospitalisation | Hospital-presenting substance use | Cousin comparison | 0.00000 |
| Parental psychiatric hospitalisation | Psychotropic medication purchase | Cousin comparison | 0.00000 |
| Parental psychiatric hospitalisation | Two-year NEET | Cousin comparison | 0.00000 |
| Parental psychiatric hospitalisation | Violent crime arrest | Cousin comparison | 0.00256 |
| Parental psychiatric hospitalisation | Property crime arrest | Cousin comparison | 0.00000 |
| Parental substance-attributable hospitalisation | Hospital-presenting psychiatric disorder | Crude model, population | 0.00000 |
| Parental substance-attributable hospitalisation | Hospital-presenting substance use | Crude model, population | 0.00000 |
| Parental substance-attributable hospitalisation | Psychotropic medication purchase | Crude model, population | 0.00000 |
| Parental substance-attributable hospitalisation | Two-year NEET | Crude model, population | 0.00000 |
| Parental substance-attributable hospitalisation | Violent crime arrest | Crude model, population | 0.00000 |
| Parental substance-attributable hospitalisation | Property crime arrest | Crude model, population | 0.00000 |
| Parental substance-attributable hospitalisation | Hospital-presenting psychiatric disorder | Adjusted model, population | 0.00000 |
| Parental substance-attributable hospitalisation | Hospital-presenting substance use | Adjusted model, population | 0.00000 |
| Parental substance-attributable hospitalisation | Psychotropic medication purchase | Adjusted model, population | 0.00000 |
| Parental substance-attributable hospitalisation | Two-year NEET | Adjusted model, population | 0.00000 |
| Parental substance-attributable hospitalisation | Violent crime arrest | Adjusted model, population | 0.00000 |
| Parental substance-attributable hospitalisation | Property crime arrest | Adjusted model, population | 0.00000 |
| Parental substance-attributable hospitalisation | Hospital-presenting psychiatric disorder | Cousin comparison | 0.00000 |
| Parental substance-attributable hospitalisation | Hospital-presenting substance use | Cousin comparison | 0.00000 |
| Parental substance-attributable hospitalisation | Psychotropic medication purchase | Cousin comparison | 0.00000 |
| Parental substance-attributable hospitalisation | Two-year NEET | Cousin comparison | 0.00000 |
| Parental substance-attributable hospitalisation | Violent crime arrest | Cousin comparison | 0.00000 |
| Parental substance-attributable hospitalisation | Property crime arrest | Cousin comparison | 0.00000 |
| Parental death | Hospital-presenting psychiatric disorder | Crude model, population | 0.00000 |
| Parental death | Hospital-presenting substance use | Crude model, population | 0.00000 |
| Parental death | Psychotropic medication purchase | Crude model, population | 0.00000 |
| Parental death | Two-year NEET | Crude model, population | 0.00000 |
| Parental death | Violent crime arrest | Crude model, population | 0.00000 |
| Parental death | Property crime arrest | Crude model, population | 0.00000 |
| Parental death | Hospital-presenting psychiatric disorder | Adjusted model, population | 0.00000 |
| Parental death | Hospital-presenting substance use | Adjusted model, population | 0.00000 |
| Parental death | Psychotropic medication purchase | Adjusted model, population | 0.00000 |
| Parental death | Two-year NEET | Adjusted model, population | 0.00000 |
| Parental death | Violent crime arrest | Adjusted model, population | 0.00000 |
| Parental death | Property crime arrest | Adjusted model, population | 0.00000 |
| Parental death | Hospital-presenting psychiatric disorder | Cousin comparison | 0.00000 |
| Parental death | Hospital-presenting substance use | Cousin comparison | 0.01578 |
| Parental death | Psychotropic medication purchase | Cousin comparison | 0.00001 |
| Parental death | Two-year NEET | Cousin comparison | 0.00283 |
| Parental death | Violent crime arrest | Cousin comparison | 0.00094 |
| Parental death | Property crime arrest | Cousin comparison | 0.00003 |
| Parental prison sentence | Hospital-presenting psychiatric disorder | Crude model, population | 0.00000 |
| Parental prison sentence | Hospital-presenting substance use | Crude model, population | 0.00000 |
| Parental prison sentence | Psychotropic medication purchase | Crude model, population | 0.00000 |
| Parental prison sentence | Two-year NEET | Crude model, population | 0.00000 |
| Parental prison sentence | Violent crime arrest | Crude model, population | 0.00000 |
| Parental prison sentence | Property crime arrest | Crude model, population | 0.00000 |
| Parental prison sentence | Hospital-presenting psychiatric disorder | Adjusted model, population | 0.00000 |
| Parental prison sentence | Hospital-presenting substance use | Adjusted model, population | 0.00000 |
| Parental prison sentence | Psychotropic medication purchase | Adjusted model, population | 0.00000 |
| Parental prison sentence | Two-year NEET | Adjusted model, population | 0.00000 |
| Parental prison sentence | Violent crime arrest | Adjusted model, population | 0.00000 |
| Parental prison sentence | Property crime arrest | Adjusted model, population | 0.00000 |
| Parental prison sentence | Hospital-presenting psychiatric disorder | Cousin comparison | 0.00000 |
| Parental prison sentence | Hospital-presenting substance use | Cousin comparison | 0.00368 |
| Parental prison sentence | Psychotropic medication purchase | Cousin comparison | 0.00006 |
| Parental prison sentence | Two-year NEET | Cousin comparison | 0.00000 |
| Parental prison sentence | Violent crime arrest | Cousin comparison | 0.00000 |
| Parental prison sentence | Property crime arrest | Cousin comparison | 0.00001 |
| Parental union dissolution | Hospital-presenting psychiatric disorder | Crude model, population | 0.00000 |
| Parental union dissolution | Hospital-presenting substance use | Crude model, population | 0.00000 |
| Parental union dissolution | Psychotropic medication purchase | Crude model, population | 0.00000 |
| Parental union dissolution | Two-year NEET | Crude model, population | 0.00000 |
| Parental union dissolution | Violent crime arrest | Crude model, population | 0.00000 |
| Parental union dissolution | Property crime arrest | Crude model, population | 0.00000 |
| Parental union dissolution | Hospital-presenting psychiatric disorder | Adjusted model, population | 0.00000 |
| Parental union dissolution | Hospital-presenting substance use | Adjusted model, population | 0.00000 |
| Parental union dissolution | Psychotropic medication purchase | Adjusted model, population | 0.00000 |
| Parental union dissolution | Two-year NEET | Adjusted model, population | 0.00000 |
| Parental union dissolution | Violent crime arrest | Adjusted model, population | 0.00000 |
| Parental union dissolution | Property crime arrest | Adjusted model, population | 0.00000 |
| Parental union dissolution | Hospital-presenting psychiatric disorder | Cousin comparison | 0.00000 |
| Parental union dissolution | Hospital-presenting substance use | Cousin comparison | 0.00000 |
| Parental union dissolution | Psychotropic medication purchase | Cousin comparison | 0.00000 |
| Parental union dissolution | Two-year NEET | Cousin comparison | 0.00000 |
| Parental union dissolution | Violent crime arrest | Cousin comparison | 0.00000 |
| Parental union dissolution | Property crime arrest | Cousin comparison | 0.00000 |
| Parental social assistance | Hospital-presenting psychiatric disorder | Crude model, population | 0.00000 |
| Parental social assistance | Hospital-presenting substance use | Crude model, population | 0.00000 |
| Parental social assistance | Psychotropic medication purchase | Crude model, population | 0.00000 |
| Parental social assistance | Two-year NEET | Crude model, population | 0.00000 |
| Parental social assistance | Violent crime arrest | Crude model, population | 0.00000 |
| Parental social assistance | Property crime arrest | Crude model, population | 0.00000 |
| Parental social assistance | Hospital-presenting psychiatric disorder | Adjusted model, population | 0.00000 |
| Parental social assistance | Hospital-presenting substance use | Adjusted model, population | 0.00000 |
| Parental social assistance | Psychotropic medication purchase | Adjusted model, population | 0.00000 |
| Parental social assistance | Two-year NEET | Adjusted model, population | 0.00000 |
| Parental social assistance | Violent crime arrest | Adjusted model, population | 0.00000 |
| Parental social assistance | Property crime arrest | Adjusted model, population | 0.00000 |
| Parental social assistance | Hospital-presenting psychiatric disorder | Cousin comparison | 0.00000 |
| Parental social assistance | Hospital-presenting substance use | Cousin comparison | 0.00000 |
| Parental social assistance | Psychotropic medication purchase | Cousin comparison | 0.00000 |
| Parental social assistance | Two-year NEET | Cousin comparison | 0.00000 |
| Parental social assistance | Violent crime arrest | Cousin comparison | 0.00000 |
| Parental social assistance | Property crime arrest | Cousin comparison | 0.00000 |
| One indicator | Hospital-presenting psychiatric disorder | Adjusted model, population | 0.00000 |
| Two indicators | Hospital-presenting psychiatric disorder | Adjusted model, population | 0.00000 |
| Three indicators | Hospital-presenting psychiatric disorder | Adjusted model, population | 0.00000 |
| Four or more indicators | Hospital-presenting psychiatric disorder | Adjusted model, population | 0.00000 |
| One indicator | Hospital-presenting psychiatric disorder | Cousin comparison | 0.00000 |
| Two indicators | Hospital-presenting psychiatric disorder | Cousin comparison | 0.00000 |
| Three indicators | Hospital-presenting psychiatric disorder | Cousin comparison | 0.00000 |
| Four or more indicators | Hospital-presenting psychiatric disorder | Cousin comparison | 0.00000 |
| One indicator | Hospital-presenting substance use | Adjusted model, population | 0.00000 |
| Two indicators | Hospital-presenting substance use | Adjusted model, population | 0.00000 |
| Three indicators | Hospital-presenting substance use | Adjusted model, population | 0.00000 |
| Four or more indicators | Hospital-presenting substance use | Adjusted model, population | 0.00000 |
| One indicator | Hospital-presenting substance use | Cousin comparison | 0.00000 |
| Two indicators | Hospital-presenting substance use | Cousin comparison | 0.00000 |
| Three indicators | Hospital-presenting substance use | Cousin comparison | 0.00000 |
| Four or more indicators | Hospital-presenting substance use | Cousin comparison | 0.00000 |
| One indicator | Psychotropic medication purchase | Adjusted model, population | 0.00000 |
| Two indicators | Psychotropic medication purchase | Adjusted model, population | 0.00000 |
| Three indicators | Psychotropic medication purchase | Adjusted model, population | 0.00000 |
| Four or more indicators | Psychotropic medication purchase | Adjusted model, population | 0.00000 |
| One indicator | Psychotropic medication purchase | Cousin comparison | 0.00000 |
| Two indicators | Psychotropic medication purchase | Cousin comparison | 0.00000 |
| Three indicators | Psychotropic medication purchase | Cousin comparison | 0.00000 |
| Four or more indicators | Psychotropic medication purchase | Cousin comparison | 0.00000 |
| One indicator | Two-year NEET | Adjusted model, population | 0.00000 |
| Two indicators | Two-year NEET | Adjusted model, population | 0.00000 |
| Three indicators | Two-year NEET | Adjusted model, population | 0.00000 |
| Four or more indicators | Two-year NEET | Adjusted model, population | 0.00000 |
| One indicator | Two-year NEET | Cousin comparison | 0.00000 |
| Two indicators | Two-year NEET | Cousin comparison | 0.00000 |
| Three indicators | Two-year NEET | Cousin comparison | 0.00000 |
| Four or more indicators | Two-year NEET | Cousin comparison | 0.00000 |
| One indicator | Violent crime arrest | Adjusted model, population | 0.00000 |
| Two indicators | Violent crime arrest | Adjusted model, population | 0.00000 |
| Three indicators | Violent crime arrest | Adjusted model, population | 0.00000 |
| Four or more indicators | Violent crime arrest | Adjusted model, population | 0.00000 |
| One indicator | Violent crime arrest | Cousin comparison | 0.00000 |
| Two indicators | Violent crime arrest | Cousin comparison | 0.00000 |
| Three indicators | Violent crime arrest | Cousin comparison | 0.00000 |
| Four or more indicators | Violent crime arrest | Cousin comparison | 0.00000 |
| One indicator | Property crime arrest | Adjusted model, population | 0.00000 |
| Two indicators | Property crime arrest | Adjusted model, population | 0.00000 |
| Three indicators | Property crime arrest | Adjusted model, population | 0.00000 |
| Four or more indicators | Property crime arrest | Adjusted model, population | 0.00000 |
| One indicator | Property crime arrest | Cousin comparison | 0.00000 |
| Two indicators | Property crime arrest | Cousin comparison | 0.00000 |
| Three indicators | Property crime arrest | Cousin comparison | 0.00000 |
| Four or more indicators | Property crime arrest | Cousin comparison | 0.00000 |
| All p-values adjusted for False Discovery rate (FDR) | | | |

Supplementary table S12: Attenuation between population-level covariate-adjusted models and cousin comparisons and average attenuation across all exposures and outcomes.

|  | Offspring outcomes | | | | | | |
| --- | --- | --- | --- | --- | --- | --- | --- |
|  | Hospital-presenting psychiatric disorder | Hospital-presenting substance use | Psychotropic medication purchases | Two-year NEET | Violent crime arrest | Property crime arrest | Average attenuation across outcomes |
| Household dysfunction indicators  (measured at child's ages 0-14) | Attenuation % | Attenuation % | Attenuation % | Attenuation % | Attenuation % | Attenuation % | Attenuation % |
| Parental psychiatric hospitalisation | 6 | 6 | **11** | 6 | 15 | 13 | 9 |
| Parental substance-attributable hospitalisation | 7 | 13 | 8 | 4 | 12 | **21** | 11 |
| Parental death | 4 | 19 | 1 | 6 | -2 | 1 | 5 |
| Parental prison sentence | 1 | **39** | **17** | 10 | **30** | **38** | 22 |
| Parental union dissolution | 6 | **21** | **7** | **11** | **14** | **21** | 13 |
| Parental social assistance | 9 | **15** | **9** | **12** | **14** | **22** | 14 |
| Average attenuation across exposures | 6 | 19 | 9 | 8 | 14 | 19 | 12 |
| Average attenuation of all associations (range): 12% (-2,39)  Comparisons where 95% confidence intervals in population-level covariate-adjusted model and cousin comparison do not overlap are in bold  NEET: Not in education, employment, or training | | | | | | | |

Supplementary table S13: Associations between the sum score of indicators of household dysfunction and psychiatric, criminal and social outcomes in the total population and in the cousin comparison, and percentage attenuated between the population model and the cousin comparison.

|  | Hospital-presenting psychiatric disorder | Hospital-presenting substance use | Psychotropic medication purchases | Two-year NEET | Violent crime arrest | Property crime arrest |
| --- | --- | --- | --- | --- | --- | --- |
| **Population-level model (n=835 987)** |  |  |  |  |  |  |
| Number of indicators (ref. none) | HR [95% CI] | HR [95% CI] | HR [95% CI] | HR [95% CI] | HR [95% CI] | HR [95% CI] |
| One | 1.48 [1.46,1.50] | 1.63 [1.59,1.67] | 1.36 [1.34,1.38] | 1.43 [1.41,1.45] | 1.63 [1.59,1.67] | 1.63 [1.60,1.66] |
| Two | 2.02 [1.99,2.05] | 2.41 [2.34,2.48] | 1.77 [1.75,1.80] | 1.87 [1.85,1.90] | 2.41 [2.35,2.48] | 2.46 [2.41,2.50] |
| Three | 2.58 [2.52,2.64] | 3.28 [3.16,3.41] | 2.16 [2.12,2.21] | 2.14 [2.09,2.18] | 3.08 [2.98,3.19] | 3.09 [3.01,3.18] |
| Four or more | 3.00 [2.92,3.09] | 4.33 [4.15,4.52] | 2.50 [2.44,2.56] | 2.37 [2.31,2.43] | 3.66 [3.52,3.81] | 3.60 [3.49,3.72] |
| **Cousin comparison (n=87 500)** |  |  |  |  |  |  |
| Number of indicators (ref. none) | HR [95% CI] | HR [95% CI] | HR [95% CI] | HR [95% CI] | HR [95% CI] | HR [95% CI] |
| One | 1.40 [1.31,1.50] | 1.56 [1.37,1.78] | 1.27 [1.20,1.34] | 1.27 [1.20,1.35] | 1.47 [1.30,1.65] | 1.35 [1.24,1.47] |
| Two | 1.89 [1.75,2.05] | 2.00 [1.73,2.31] | 1.70 [1.59,1.81] | 1.61 [1.50,1.72] | 2.04 [1.78,2.32] | 1.85 [1.68,2.04] |
| Three | 2.29 [2.02,2.59] | 2.55 [2.06,3.16] | 1.77 [1.59,1.96] | 1.80 [1.61,2.01] | 2.56 [2.10,3.13] | 2.04 [1.76,2.36] |
| Four or more | 2.97 [2.54,3.48] | 3.10 [2.41,3.97] | 2.27 [1.98,2.60] | 2.17 [1.89,2.50] | 2.67 [2.11,3.38] | 2.39 [2.00,2.84] |
| **Attenuation between population-level model and cousin comparison** |  | |  |  |  |  |
| Number of indicators | % attenuated | % attenuated | % attenuated | % attenuated | % attenuated | % attenuated |
| One (average attenuation 8%) | 5 | 4 | **7** | **11** | 10 | **17** |
| Two (average attenuation 14%) | 6 | **17** | 4 | **14** | **16** | **25** |
| Three (average attenuation 20%) | 11 | **22** | **18** | **16** | 17 | **34** |
| Four or more (average attenuation 18%) | 1 | **28** | 9 | 8 | **27** | **34** |
| Average attenuation across categories: | 6 | 18 | 10 | 12 | 17 | 27 |
| NEET: Not in education, employment or training  Models are adjusted for parental education, region of residence, birth order, maternal age, child’s sex and an indicator for two-parent family at age 0.  In the attenuation percentages, comparisons where 95% confidence intervals in population-level covariate-adjusted model and cousin comparison do not overlap are in bold (calculated before rounding) | | | | | | |

Supplementary table S14: Number and proportion of individuals excluded from the total in the sensitivity analyses including only incident outcomes

|  | Total | Previous hospital-presenting psychiatric disorder | Previous hospital-presenting substance use | Previous psychotropic medication purchase | Previous violent crime | Previous property crime |
| --- | --- | --- | --- | --- | --- | --- |
|  | n | n [%] | n [%] | n [%] | n [%] | n [%] |
| Total population | 835 987 | 20 573 [2] | 4390 [1] | 64 270 [8] | 6061 [1] | 41 865 [5] |
| Cousin population | 87 500 | 2088 [2] | 345 [0] | 7096 [8] | 478 [1] | 3491 [4] |
| Note: NEET (not in education, employment or training) could only occur after age 14 and therefore no one could be NEET before the start of follow-up | | | | | | |

Supplementary table S15: Tetrachoric correlations between the childhood household dysfunction indicators in the total population (n=835 987)

Supplementary table S16: Results from an exploratory factor analysis on a tetrachoric correlation matrix of the childhood household dysfunction indicators

| Eigenvalues of the factors | | |
| --- | --- | --- |
| Factor | Eigenvalue | % of the total variance explained |
| F1 | 2.90 | 0.94 |
| F2 | 0.37 | 0.12 |
| F3 | 0.20 | 0.07 |
| F4 | -0.07 | -0.02 |
| F5 | -0.10 | -0.03 |
| F6 | -0.22 | -0.07 |
| Factor loadings on variables | | |
| Variable | F1 | Uniqueness |
| Parental psychiatric hospitalisation | 0.67 | 0.56 |
| Parental substance-attributable hospitalisation | 0.85 | 0.27 |
| Parental death | 0.46 | 0.79 |
| Parental prison sentence | 0.75 | 0.43 |
| Parental union dissolution | 0.58 | 0.66 |
| Parental social assistance | 0.78 | 0.39 |
| Only factors fulfilling the Kaiser criterion (eigenvalue>1) retained | | |

|  | Parental psychiatric hospitalisation | Parental substance-attributable hospitalisation | Parental death | Parental prison sentence | Parental union dissolution | Parental social assistance |
| --- | --- | --- | --- | --- | --- | --- |
| Parental psychiatric hospitalisation | 1.00 |  |  |  |  |  |
| Parental substance-attributable hospitalisation | 0.69 | 1.00 |  |  |  |  |
| Parental death | 0.32 | 0.50 | 1.00 |  |  |  |
| Parental prison sentence | 0.37 | 0.64 | 0.36 | 1.00 |  |  |
| Parental union dissolution | 0.38 | 0.46 | 0.12 | 0.40 | 1.00 |  |
| Parental social assistance | 0.46 | 0.56 | 0.29 | 0.70 | 0.58 | 1.00 |

Supplementary figure S1: Proportions of the total sum of household dysfunction indicators accounted by each specific indicator across the categories of the sum score variable.


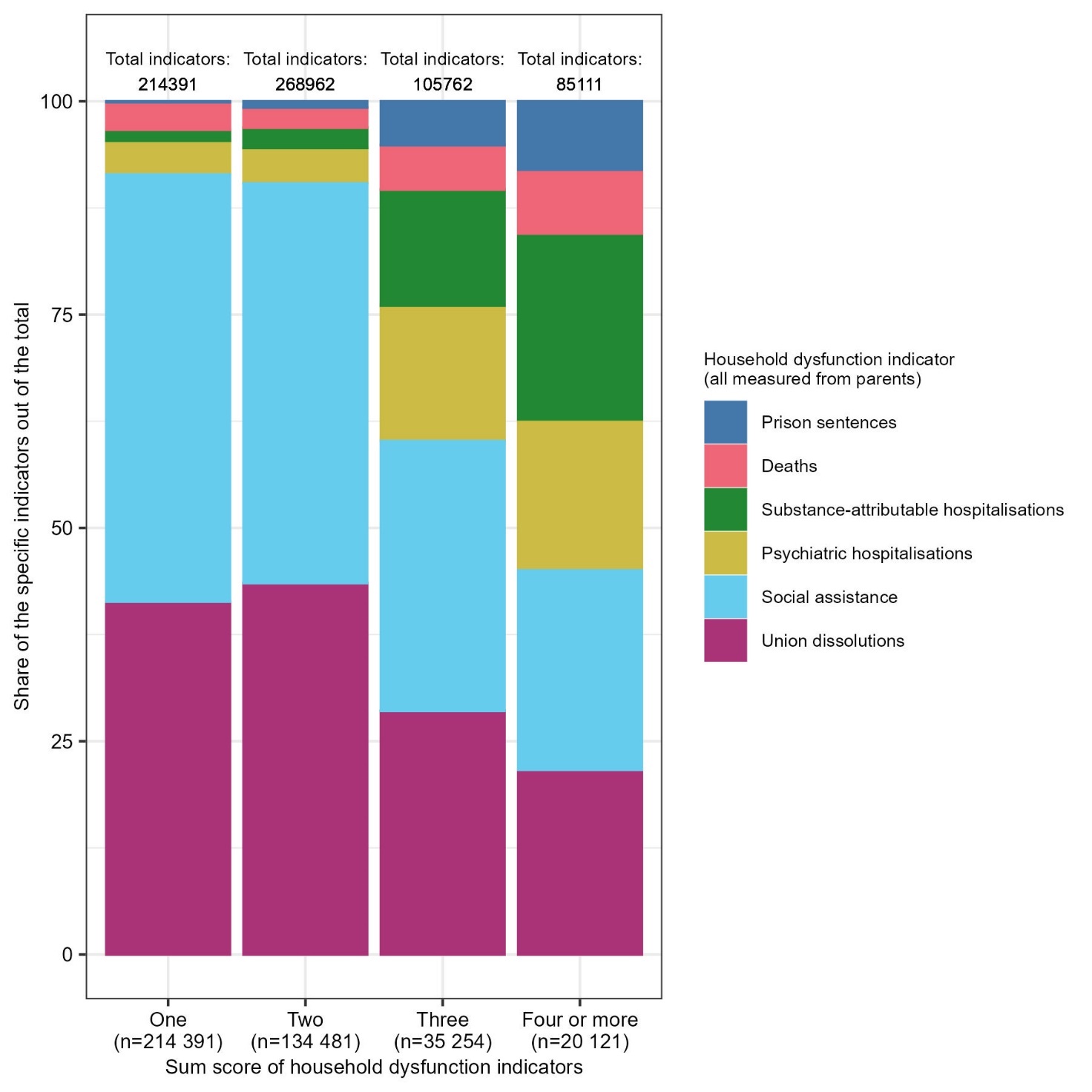


Supplementary figure S2: Associations between single indicators of household dysfunction and psychiatric and criminal outcomes in emerging adulthood in crude and confounder-adjusted models in the total population and in confounder-adjusted model in the cousin comparison. Results from the main analysis and sensitivity analysis excluding prevalent outcomes.


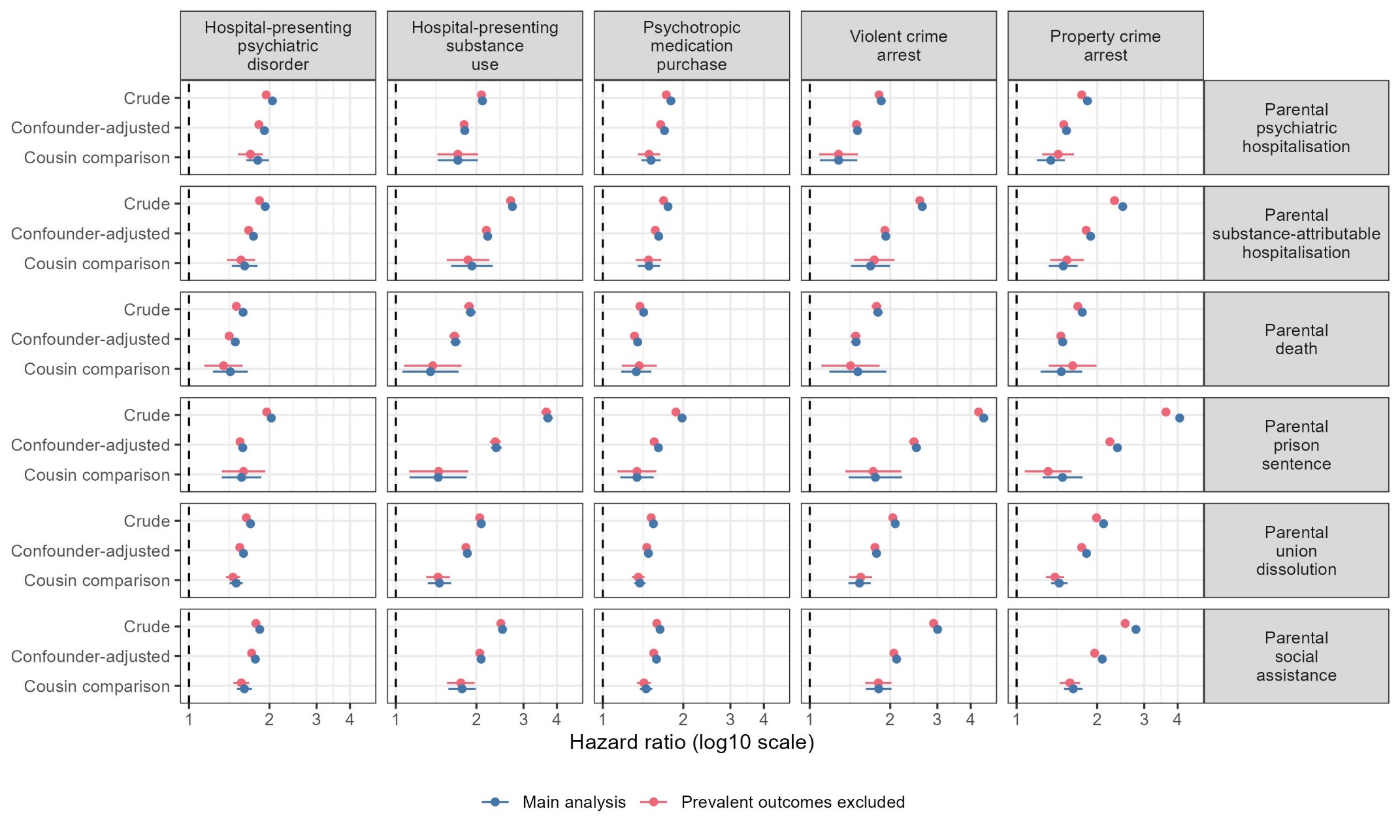
 Note: Not in education employment or training not included as no one were NEET before start of follow-up.

Supplementary figure S3: Associations between the sum score of indicators of household dysfunction and psychiatric and criminal outcomes in the total population and in the cousin comparison. Results from the main analysis and sensitivity analysis excluding prevalent outcomes.


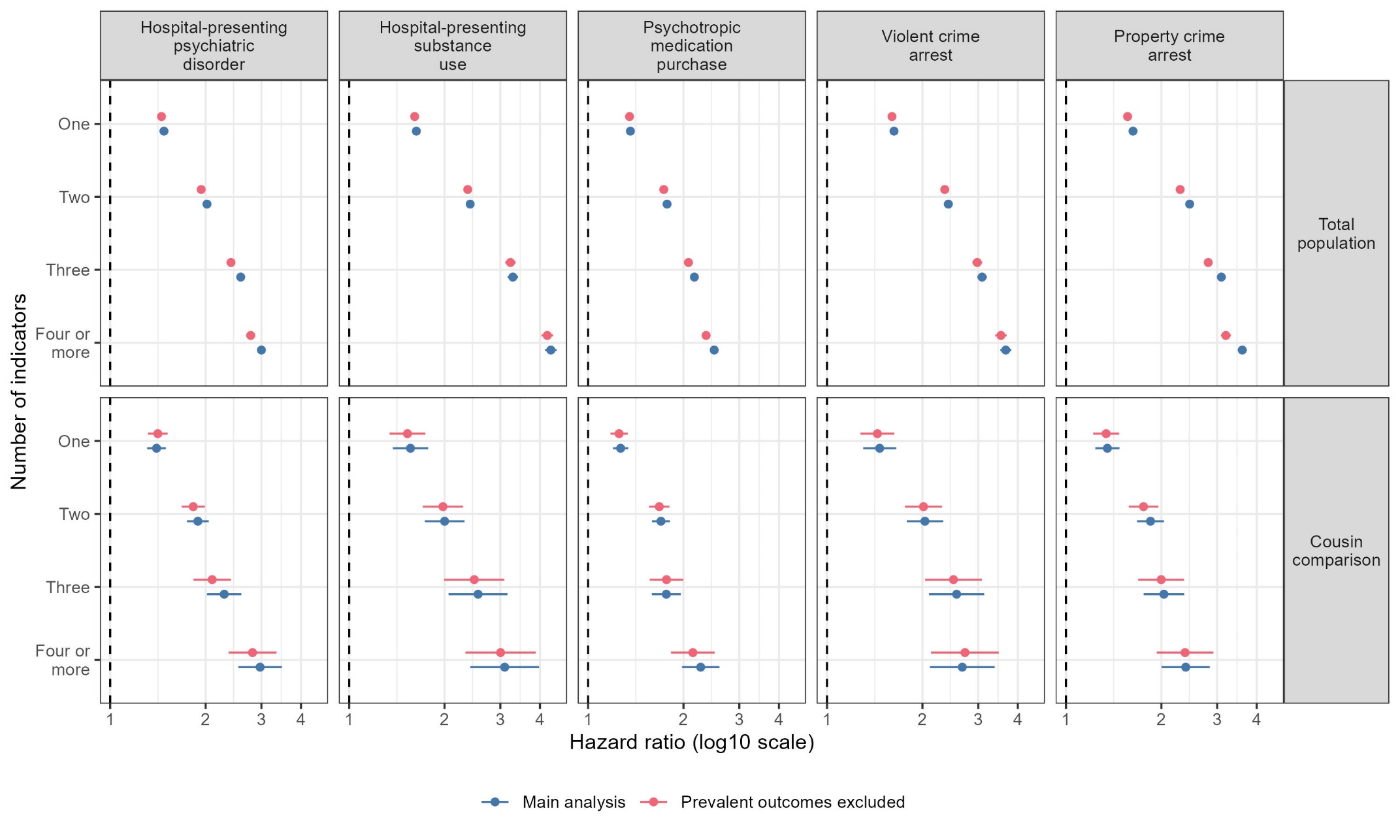


Note: Not in education employment or training not included as no one were NEET before start of follow-up.
